# Analysis of Organic Residues on Neolithic Pottery in Different Settlements in Poland

**DOI:** 10.3390/molecules31132309

**Published:** 2026-07-01

**Authors:** Łukasz Orszański, Angelina Rosiak, Joanna Sekulska-Jaworska, Jarosław Gocławski, Joanna Kałużna-Czaplińska

**Affiliations:** 1 Institute of General and Ecological Chemistry, Faculty of Chemistry, Lodz University of Technology, Żeromskiego 114, 90-543 Lodz, Poland; lukasz.orszanski@dokt.p.lodz.pl; 2 Institute of Applied Computer Science, Faculty of Electrical, Electronic, Computer and Control Engineering, Lodz University of Technology, Stefanowskiego 18, 90-537 Lodz, Poland; joanna.sekulska-jaworska@p.lodz.pl (J.S.-J.); jaroslaw.goclawski@p.lodz.pl (J.G.)

**Keywords:** chromatographic analyses, archaeological ceramics, gas chromatography–mass spectrometry, unsupervised learning, PERMANOVA

## Abstract

Chemical analysts and archeologists are increasingly interested in organic remains that penetrate the porous structures of ceramic vessels. Fatty acids and archaeological biomarkers are chemical compounds that are particularly important for determining the contents of ceramic vessels. This study involved gas chromatography coupled with mass spectrometry (GC–MS) analysis of organic residues extracted from 56 Neolithic pottery samples found in 18 different settlements in Poland. Fatty acid ratios, including the newly proposed C15:0/C17:0 ratio (pentadecanoic acid/heptadecanoic acid) for the identification of dairy products and archaeological biomarker analysis, were used to determine the possible origin of these residues. The data obtained from the gas chromatography studies were statistically analyzed using principal component analysis (PCA), k-means clustering, and PERMANOVA to determine differences in the diet of the people inhabiting individual settlements. The obtained results allowed us to determine that the Neolithic diet was probably similar in different regions of Poland and throughout different periods of the Neolithic era. However, because of the large difference in variance between the different sample groups, we believe that research should continue and that a larger number of samples per settlement or historical period should be examined. We can conclude that all samples contained residues of mixed animal and plant origin, and the food stored in these vessels was likely subjected to thermal processing.

## 1. Introduction

The study of organic residues in prehistoric pottery is particularly important for determining the diet of our ancestors and the use of the studied vessels. Organic residues from food may still be present inside the vessels in the form of content, they may cover the vessels in the form of layers (e.g., carbon deposits), or they may be absorbed into the porous structure of clay vessels. The latter constitutes the largest group of preserved organic residues (found in approximately 80% of preserved clay vessels [1]) and can be extracted and analyzed in the laboratory using different extraction and chromatographic methods [2,3]. The porous structure of clay protects the food inside from microbial degradation and external conditions, even when the vessels are buried for hundreds or thousands of years. The chemical compounds present in organic residue tests originate from many different sources. These may be animal-derived residues, plant-derived residues, organic compounds resulting from the thermal processing of vessels and their contents, and contaminants.

Lipids, including fatty acids, waxes, and sterols [4], are a special group of compounds used to determine the origin of organic residues because they are relatively stable compared to proteins or DNA, and are therefore easy to identify in the samples tested [2]. Another group of compounds that are of great importance in archaeometry are the so-called archaeological biomarkers, i.e., substances characteristic of a given type of food [1]. Compounds such as cholesterol or phytosterols allow us to unambiguously determine whether organic residues are of animal or plant origin. The presence of waxes and resins may indicate attempts to seal vessels [5], whereas the presence of sugars and tartaric acid may indicate wine or other alcoholic beverages [6].

Chromatographic tests using gas and liquid chromatography are particularly important in the study of prehistoric ceramics, as they allow for a thorough analysis of the chemical composition of samples. The most commonly used methods in archaeometry include gas chromatography coupled with mass spectroscopy (GC–MS), gas chromatography–combustion–isotope ratio mass spectrometry (GC–C–IRMS), gas chromatography–time-of-flight mass spectrometry (GC–TOFMS), and liquid chromatography–mass spectrometry (LC–MS) [2]. Various methods have been developed for identifying the origins of organic residues. The most commonly used methods include lipid analysis using GC–MS and analysis of relatively stable isotopes of carbon, nitrogen, or oxygen using GC–C–IRMS [4].

One of the most important issues in the context of Neolithic pottery research is the differentiation between ruminant and non-ruminant fats and the analysis of milk processing. These studies attempt to answer the question of how animals were bred in the Neolithic period and the extent to which milk was a component of the diet of our ancestors. Currently, two approaches dominate this area: one based on stable carbon isotopes and the other on fatty acid proportions.

The first approach, based on carbon isotope (δ^13^C) studies by Dudd and Evershed, showed that milk and adipose fat from animals raised on similar pastures and fodders have distinct isotopic signatures and therefore reveal how much dairy products contributed to the diet of a given Neolithic community [7].

Several studies have shown that animals were raised for both milk and meat from the earliest days of the Neolithic period in both Europe [8,9,10,11,12] and the British Isles [13,14,15]. The first attempts at milk production probably appeared at the time of animal domestication, although the scale of this phenomenon changed over time and probably evolved towards increased milk production [8]. This was due to the initially low lactase levels in these communities, as well as many other geographical and living conditions. Evershed et al. examined pottery samples from the Near East and Eastern Europe using GC and showed that the first cases of storing and thermally processing milk in ceramic vessels occurred as early as the 5th-7th millennium BC [16]. On the other hand, GC–MS and GC–C–IRMS studies by Craig et al. on fats from wild ruminants show that residues commonly identified as milk fats may also originate from wild animals, such as deer, and this may indicate that dairy products were likely initially produced on a small scale. [17]. Another important aspect is the processing of dairy products into more durable forms by milk fermentation, such as cheese, which allows for the consumption of dairy products for a longer period of time and also reduces the amount of lactose in them [9].

When it comes to research on the Neolithic diet in Poland, it is worth mentioning the work of Evans et al., who, based on a comparison of lipid studies and protein deposits on the walls of vessels, demonstrated that lipid profile studies based on δ^13^C carbon isotope analysis may lead to an underestimation of the consumption of dairy products in the vessels studied, because milk proteins are also present in vessels where the milk lipid profile was not detected. This was probably due to the fact that fats from different foods mix together in the dishes used, which affects the fatty acid content from milk fats and adipose tissue fats [18].

The second approach for determining the origin of organic residues is based on fatty acid proportions. This is because fatty acids are relatively stable compared to other organic residues, such as proteins or DNA. The proportions most commonly used in our previous studies [3,5,19,20,21,22,23,24,25] were developed by J. W. Eerkens [26] who conducted research on ceramics from the American continent, but they are still used today to study European ceramics. Another work on the study of organic residues in ceramics was conducted by Olsson et al. They proposed a ratio to distinguish fish from mammals [27] depending on the value of the C18:0/C16:0 ratio (octadecanoic acid/hexadecanoic acid). A value below 0.48 in the presence of cholesterol can indicate that the sample contains fish organic residues. A value of >0.5 is typical for terrestrial animal lipids. As mentioned in Olsson’s paper, this ratio should also be supported by the presence of certain biomarkers, such as cholesterol and isoprenoid alkanoic acids. Hjulström et al. proposed a ratio to distinguish ruminants from non-ruminants [28]. If the value of C17:0/C18:0 (heptadecanoic acid/octadecanoic acid) is approximately >0.02, it can indicate ruminant or milk. If the value is lower than 0.0077, it is non-ruminant. Based on preliminary results, we identified a new ratio that could potentially be used to identify dairy products. The value of C15:0/C17:0 (pentadecanoic acid/heptadecanoic acid) for milk is approximately 1.0, whereas for fish, poultry, beef, pork, vegetables, and buckwheat, it is approximately 0.5. The presence of acids, such as C15:0 and C17:0, produced by microorganisms found in the rumen of ruminants can indicate ruminant meat and dairy products [29]. To date, probably the only study on the Neolithic diet in Poland using fatty acid ratios is the work on ancient ceramics found in archaeological sites of Gniechowice and Stary Zamek [30], which revealed that organic remains found in fragments of Neolithic pottery were most likely of plant origin.

The aim of our study was to demonstrate dietary habits during the Neolithic period in Polish territories based on fatty acid ratio approach and biomarker analysis. The studied vessels come from different cultures and historical periods spanning time (5200 BC–2000 BC). For this purpose, we analyzed the organic residues present in the samples tested and then performed a statistical analysis using principal component analysis (PCA) and k-means clustering to visualize the structure of both fatty acid and biomarker data. Finally, we performed PERMANOVA [31] to compare the fatty acid content in individual settlements.

## 2. Results and Discussions

### 2.1. C15:0/C17:0 Fatty Acid Ratio Analysis

In our search for a new ratio for identifying dairy products, we were guided by the fact that C15:0 is found in milk fat and ruminant meat [32]. C17:0 occurs in ruminant fat and dairy products [33]. Since both acids occur in animal products, they were selected for further study of their proportions. As part of our research on experimental vessels, we divided the food products into four groups: milk, fish, meat, and vegetables. We then calculated the fatty acid composition for each group. Consequently, we obtained a C15:0/C17:0 ratio of 0.93 for milk, 0.47 for fish, 0.59 for meat, and 0.56 for vegetables. Because the value obtained for milk differed significantly from the others (at a ratio of 1/0.5), we decided to select this ratio for further analysis of the archaeological samples.

### 2.2. Fatty Acid Analysis

In our analysis of the results, we adopted the principle that Eerkens ratios are used in such a way that if three out of four ratio values (in the fresh state) are consistent with the origin of residues, then this product was chosen in our results. Additionally, this criterion is supplemented by the proportion of C18:0/C16:0 to distinguish between the residues of mammals and fish and to compare them with Eerkens proportions. We also used the C17:0/C18:0 ratio to determine whether the organic residues originated from ruminants or non-ruminants. The ratio of C15:0/C17:0 was used to preliminarily estimate the likelihood that the organic residues originated from dairy products and was supplemented by biomarker analysis. The probable sources of residues (PSRs) are listed in Table 1. As can be seen in the table, considering Eerkens ratios only, we can say that organic residues are of mixed nature, both plant- and animal-based products. The division of food products according to the Eerkens ratios can be presented as follows: seeds and nuts (45.78%), animal-based products (27.71%), roots and tubers (13.25%), fish (9.64%), and fruits (3.61%). Interestingly, in the case of Olsson’s ratio (C18:0/C16:0), the majority (41 samples) of residues were identified as animal-based products. In 13 samples, fish was indicated. In the case of Hjulström’s ratio (C17:0/C18:0), the majority (36 samples) of residues were identified as ruminant or milk. No non-ruminants were indicated, and 23 samples did not meet the expected values of this ratio. The conclusions that can be drawn from Olsson’s proportion are supported only by the presence of cholesterol in sample 17. This can be evidence of the presence of fish residues, but it can also be a result of contamination. There is a clear discrepancy between the results obtained by Eerkens and Olsson’s ratios. Mammalian residues were consistently detected in 21 samples. In no case did both methods indicate fish remains, although they appeared in different samples in both cases. However, it should be noted that Eerkens’s ratios were developed for the American continent, and Olsson’s ratio was developed specifically for identifying fish residues in Europe. Although previously mentioned studies of Neolithic pottery in Poland indicate the dominant presence of plant residues, the discovery of bones and stone blades found in Neolithic archaeological excavations indicates beyond any doubt the presence of farm animals and meat consumption [30]. In the case of the C15:0/C17:0 ratio, the probability of occurrence of dairy products was indicated in samples 7, 9 and 38. This is supported by the presence of lactic acid in samples 7 and 9. This is also consistent with Hjulström’s ratio, which suggests that these samples can contain ruminant or milk residues.

### 2.3. Biomarker Analysis

The archaeological biomarkers detected in individual samples are summarized in Table 2 (data were available for biomarker analysis—raw chromatographic data including chromatograms and their MS spectra—for samples 1–23 and 40–56).

Cholesterol [34] and cholestane derivatives, cholest-23-ene, (5β)- and cholestan-3-ol, 2-methylene-, and (3β,5α)-, can be considered products derived from animal products. Both cholesterol and cholestane are animal sterols, but they can also be found as a result of skin contamination [35]. Squalene was found in fish oils, plant oils and human sebum [36,37]. It can be to some extent considered a fish biomarker since it occurs most abundantly in shark liver oil.

Stigmastanol, stigmasta-3,5-diene, γ-sitostenone, and γ-sitosterol, which are products of various chemical transformations of phytosterols, in particular β-sitosterol and stigmasterol [21,38], are biomarkers of plant-based products. Salicylic acid is a plant hormone [39]. Glycerol and glycerol monostearate occur in fruits and vegetables [23], are the end product of digestion in animals [24], and are also a product of lipid degradation [25]. Benzoic acid is present in beeswax [20] and propolis [40,41]. This may also be a product of the breakdown of anthocyanins by ketones in wine [42]. The presence of benzoic acid in this container may also indicate plant origin of the samples tested, as it is a component of fruits and vegetables [43]. Vanillin is a characteristic flavor component of vanilla. However, due to limited trade and the availability of local foods during the Neolithic period in Central Europe, it most likely originated from other plant sources, such as elderberries, blueberries [44], strawberries [45], and mushrooms [46]. It is also abundant in wine [47] and apple cider [48]. Vanillin may also originate from tree resin or pine dust [5]. Vanillic acid is a product of vanillin oxidation. Vanillylmandelic acid is a chemical intermediate in the synthesis of artificial vanilla flavorings [49], but it may also be one of the degradation products of vanillin or other fruit flavors. Isovanillin is an isomer of vanillin that is metabolized by aldehyde dehydrogenase to isovanillic acid [50]. Apocynin, which has a flavor similar to vanillin, can be found in a variety of plants. One of the alkanes commonly found in vanilla is eicosane [51]. Lactic acid occurs in sour milk and fermented vegetables and is produced in the skeletal muscles of mammals [52] and may be produced by the action of bacteria from malic acid, which is added to beer [53,54]. 1-dodecanol (lauryl alcohol) is a degradation product of fatty acids or a possible component of plants and waxes [23]. Dibutyl phthalate is a phthalic acid derivative. Phthalic acid and benzene may indicate the thermal processing of prepared foods (products) [23]; however, the presence of dibutyl phthalate can also be a result of contamination by plastic. Additionally, the presence of anthracene and phenanthrene, which are ingredients of carbolineum (a mixture of coal tar components), can indicate that the examined vessels were subjected to high temperatures [55]. Another explanation is the use of tars, which could be used to waterproof or repair ceramics. Phytanic acid is a fatty acid characteristic of milk fat [56]. Benzoic acid esters such as benzoic acid 2-ethylhexyl ester, benzoic acid 4-ethoxy-, ethyl ester, benzoic acid cyclohexyl ester, benzoic acid hex-3-yl ester, benzoic acid hex-3-yl ester, benzoic acid octadecyl ester and benzoic acid pentyl ester, can potentially indicate the presence of wax [57]. Methyl dehydroabietate is an indicator of resin and its products [58,59,60]. Resin may have been used to seal vessels or added to preserve, enhance, or change the flavor of wines. The presence of azelaic acid and the oxalic acid isobutyl pentadecyl ester may indicate cereal products such as wheat, rye and barley [61,62]. Fumaric acid, 4-cyanophenyl dodecyl ester, is a derivative of the biomarker of fermented beer and wort production rate [54]. Carvacrol is present in many products including thyme and marjoram [5]. 11-Eicosenoic acid is an omega-9 fatty acid found in various plant oils and nuts [63]. Arachidic acid is a constituent of many plant-based products, including cupuaçu butter [64], perilla oil [65] and peanut oil [66]. Behenic acid is abundant in plant oils, peanuts, and legumes. It can also be a potential hydrolysis product of esters in beeswax [67]. Dehydroabietic acid and isopimaric acid are widely found in many coniferous trees [68,69]. Analysis also showed the presence of camphor, which is also known as (+)-2-bornanone. Dehydroabietic acid and pimaric acid are the major components of rosin [70,71]. Gentisic acid (2,5-dihydroxybenzoic acid) is also found in wine [72]. Eugenol is found in clove oil [73] and cinnamon [74], while methyleugenol is found in various essential oils and [75] and has a significant impact on the behavior of insects towards plants [76]. Cinnamaldehyde is the substance that gives cinnamon its characteristic flavor [77]. Erythritol is found in small amounts in some fruits, including watermelons, pears, and grapes, [78] and in fungus-fermented foods [79]. Myristyl alcohol (1-tetradecanol) is also found in plant-based products such as palm kernel oil and coconut oil [80]. Taraxasterol is a triterpene alcohol found in dandelions [81]. Thymol is a component of some spices, including thyme [82]. Benzophenone occurs naturally in many plants and fungi [83]. Caryophyllene occurs widely in nature. It is a component of many plants such as pepper, melissa, ylang–ylang, marigold, and the herbs sage and basil [84]. Chrysin is a flavone found in honey, propolis, and flowers [85]. Estragole is a phenylpropene found in various plants, including fennel [86] and basil [87]. Sugars and their derivatives such as α-l-fucopyranose 1,2:3,4-bis(benzeneboronate), d-(−)-ribofuranose (isomer 2), d-arabinose, and l-(+)-rhamnose (isomer 2) can be products of the degradation of sugars present in plants, especially vegetables, nuts, and seeds. Finally, vitamin E and its derivatives such as β-tocopherol and dl-α-tocopherol were identified. The major natural sources of this vitamin are vegetable oils found in nuts, soybeans, sunflowers and corn [88].

### 2.4. Statistical Analysis

**Figure 1:**
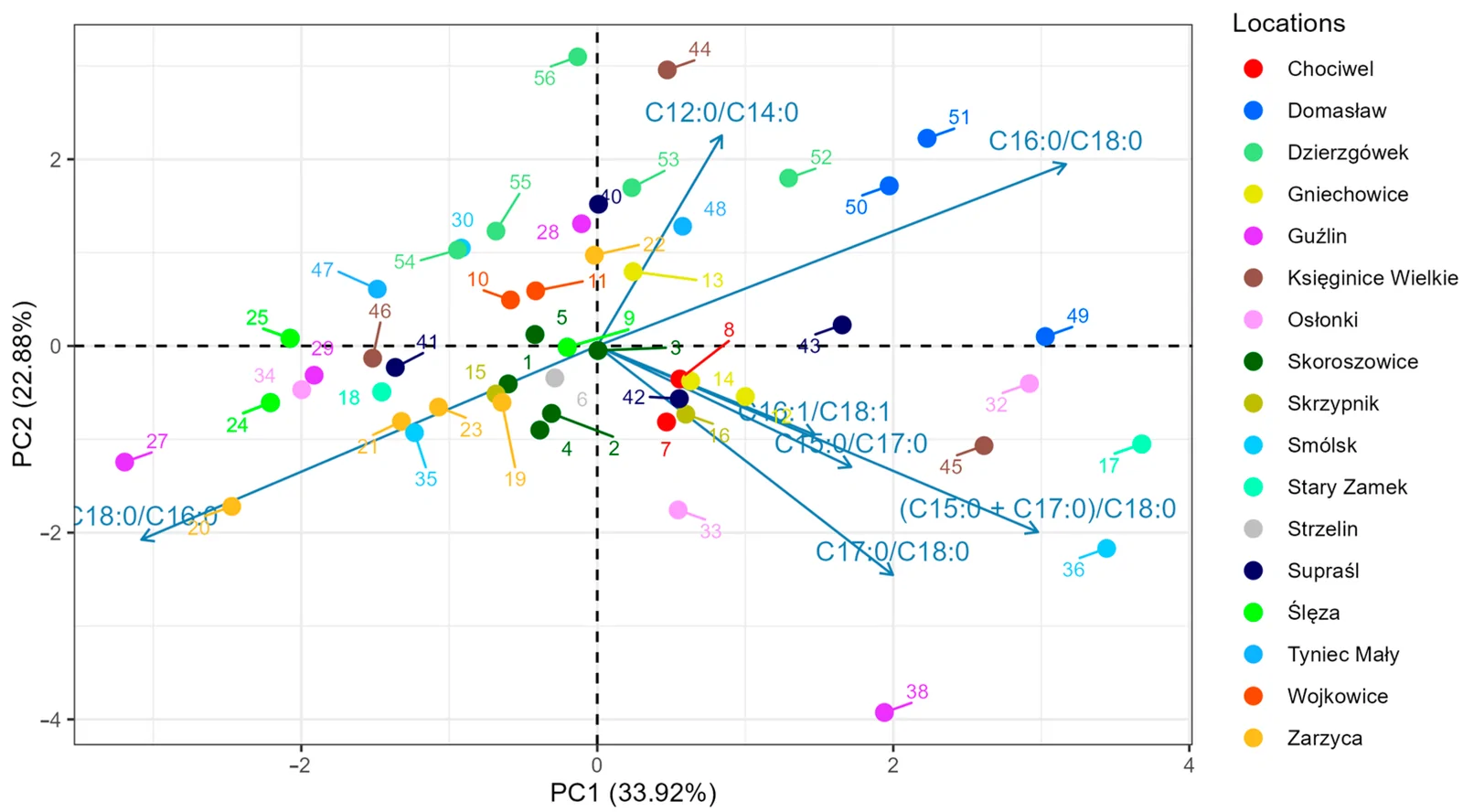
PCA of fatty acid proportions for different locations.

To investigate the structure of the fatty acid data and their variability in the context of the sampling site, we performed PCA of fatty acid proportions to evaluate whether the location of the samples could have an impact on dietary habits in these settlements. As shown in Figure 1, a few locations clearly distinguish themselves from the others. Domasław seems to be completely separated from other settlements in terms of the first and second components. The highest factor loadings for the first component were C16:0/C18:0 and (C15:0 + C17:0)/C18:0. For the second component, the highest factor loadings were found in the C12:0/C14:0 (dodecanoic acid/tetradecanoic acid) and C17:0/C18:0 ratios. Large differences in the C17:0/C18:0 and C18:0/C16:0 ratios may indicate that fish dominated the diet of the inhabitants of Domasław, distinguishing Domasław from other settlements. All other settlements are arranged in an area where there are small differences in fatty acid proportions, but they interpenetrate to a greater or lesser extent. Dzierzgówek emerged in the PCA as dependent on the second component, and the highest factor loading for this component was most likely the C12:0/C:14:0 ratio. The third component described 16.87% of the total variance (data available in the Supplementary Materials), and the observation of the data distribution confirmed the conclusions drawn from Figure 1. Additionally, it can be said that both Domasław and Dzierzgówek stand out from other settlements, with Dzierzgówek standing out in terms of the second and third components. These differences may result from the different locations of the samples studied, that is, different dietary habits in the settlements studied, as well as from the fact that they come from different historical periods. Unfortunately, in the case of samples 44–56, we do not have precise information about which specific Neolithic culture they come from; therefore, both factors resulting from location and historical period should be considered. However, in the case of samples 24–31 (5000 BC) and 32–39 (Lengyel culture), the settlements do not appear to differ significantly from the others. Some samples originating from different settlements were concentrated in the region where the Olsson (C18:0/C16:0) ratio had the greatest impact on sample diversity. The rest are scattered in the part where Hjulström (C17:0/C18:0) and Eerkens and C15:0/C17:0 ratios dominate. It is worth noting that Eerkens proportions are interpreted as a whole; therefore, it is difficult to distinguish which types of food have the greatest impact on diversity.

**Figure 2:**
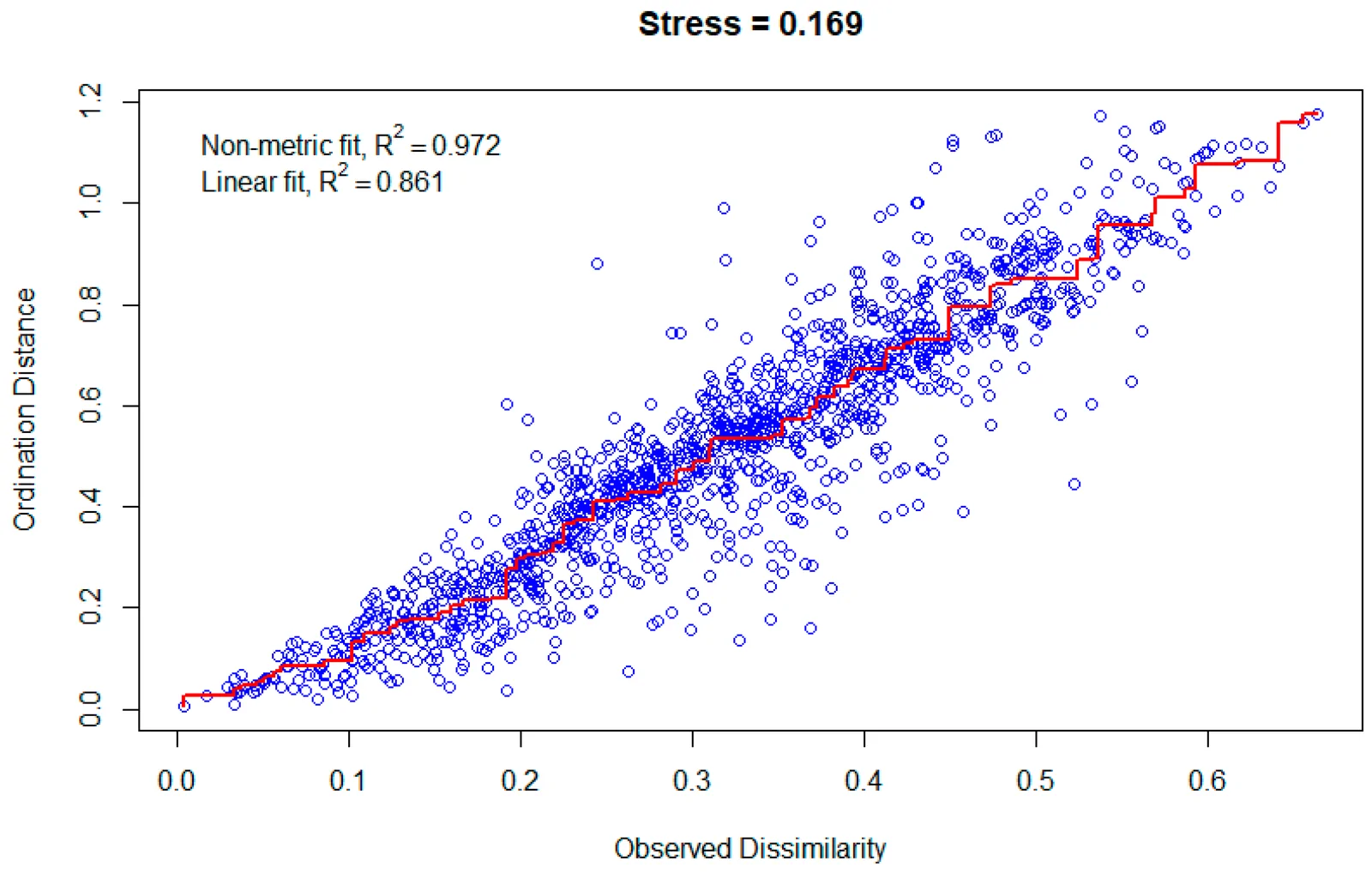
Stress plot for NMDS analysis of fatty acid proportions in different locations (blue circles represent sample pairs, the red line represents perfect agreement).

**Figure 3:**
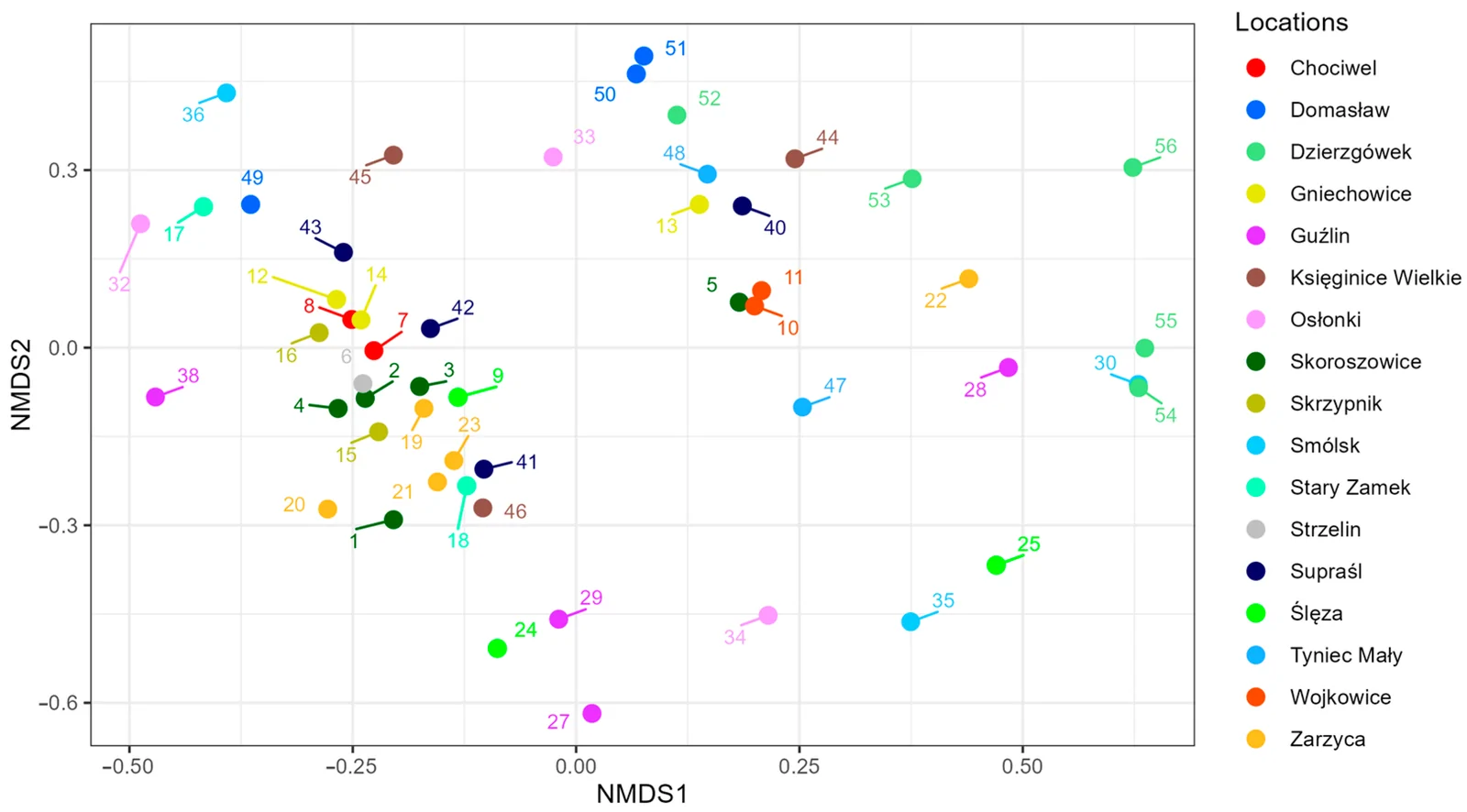
NMDS analysis of fatty acid proportions in different locations.

To determine whether individual groups of samples from different locations were significantly different from each other, we performed a multivariate analysis of variance. Because it was not possible to perform the MANOVA test due to inadequate sample sizes, that is, the number of cases in each group was smaller than the number of dependent variables, we performed PERMANOVA. Since PERMANOVA assumes homogeneity of multivariate dispersion, we visualized the data using a Non-metric MultiDimensional Scaling (NMDS) plot to evaluate whether there is a location effect of data. The stress value obtained for the NMDS analysis was approximately 0.17 (where the value of usable stress should be within the range of 0.1–0.2—Figure 2). Therefore, we can assume that the NMDS plot indicates a moderate-to-fair representation of multidimensional data. NMDS analysis confirmed the results obtained from the PCA, that is, there were differences in the distribution of fatty acid proportions at individual locations (Figure 3). To confirm that we were actually dealing with a location effect and not a dispersion effect (which could distort the PERMANOVA results), we conducted one-way ANOVA (we used *the betadisper* function from the *vegan* package to calculate the spread of the community data around the centroid and then performed the ANOVA). The *p*-value for this analysis of 0.004 (F(16, 35) = 2.96) reveals that there is a significant difference in dispersion patterns (the value is lower than the significance level of 0.05).

Although the assumption of homogeneous dispersion was violated (*p* = 0.004), we proceeded with the PERMANOVA (9999 permutations) to gain exploratory insights into potential location-based differences. We interpreted the results with an explicit understanding that the significant PERMANOVA outcome was potentially influenced by differences in group dispersion. The concomitant results from the dispersion test and the visual evidence from the NMDS plot suggest that the observed group differences are a complex function of both location and spread and should not be interpreted as a simple shift in community centroids.

The PERMANOVA revealed that the origin of samples can potentially have a statistically significant effect on dietary differences (*p* = 0.0011, F = 2.14, R^2^ = 0.49, where R^2^ is the *effect size* that indicates the proportion of total variation explained by the model). The origin of the samples explained approximately 49% of the total variation in community data.

Moreover, we conducted pairwise comparisons to evaluate which specific locations might drive the overall significance (data available in the Supplementary Materials). Pairwise comparisons were conducted using the *adonis* function with False Discovery Rate (FDR) correction for multiple testing (*n* = 125 comparisons). While several comparisons showed nominally significant differences before correction (e.g., Skoroszowice vs. Dzierzgówek: pseudo-F = 10.93, R^2^ = 0.577, *p* = 0.003), none remained significant after adjusting for multiple comparisons (all p.adjusted = 1.000; Supplementary Table S1). Effect sizes (R^2^) varied considerably across comparisons, ranging from 0.007 to 0.991, suggesting substantial heterogeneity in the degree of dissimilarity between the site pairs. Therefore, it can be concluded that no significant differences in dietary habits between individual settlements were proven.

**Figure 4:**
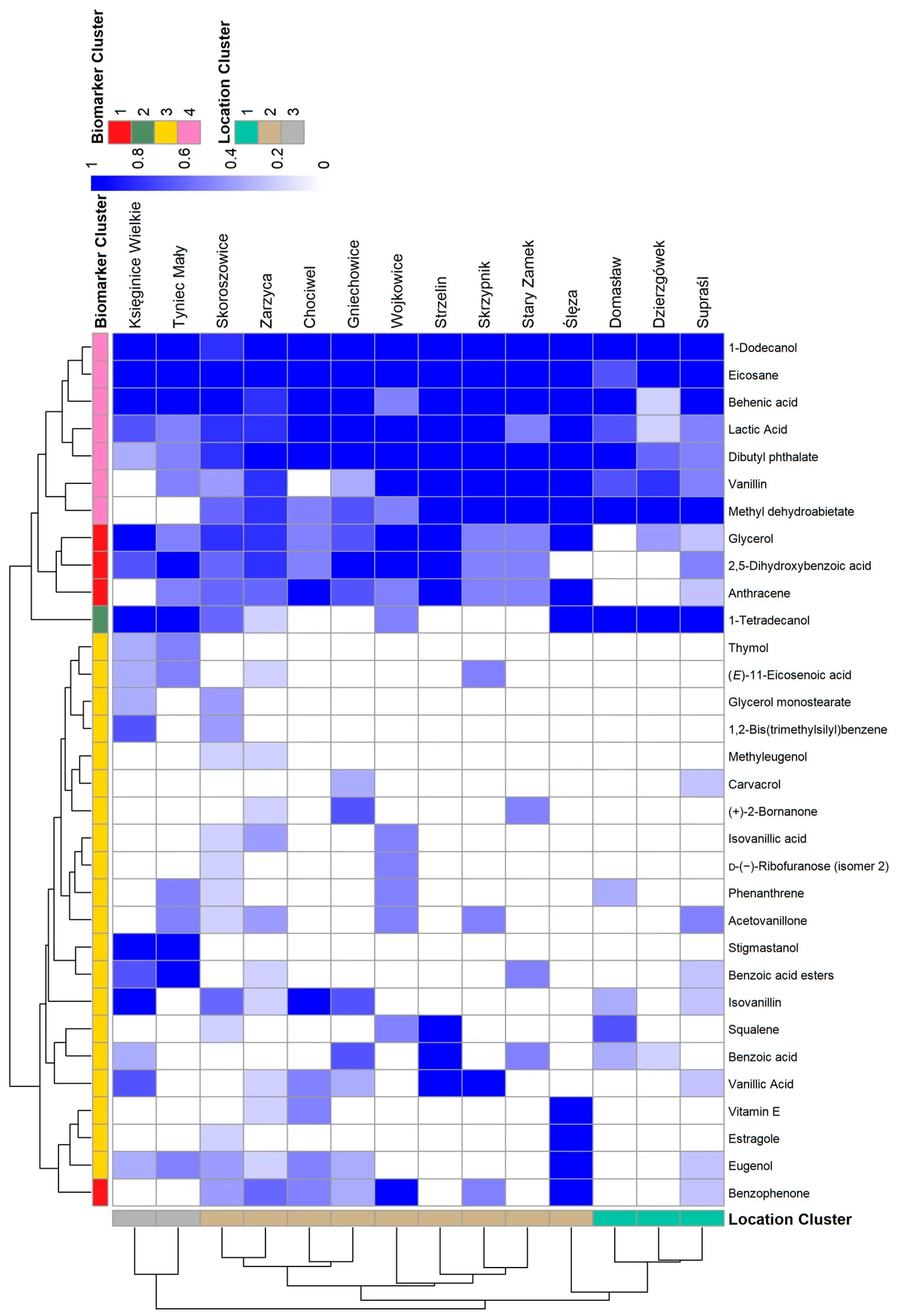
Heatmap of biomarker composition for different locations.

Regarding archaeological biomarkers, we performed clustering using the k-means algorithm and visualized the data in Figure 4 (we used the elbow method to select the best inflection point of the within-cluster sum of square metric depending on the value of the k parameter and chose k = 4 for biomarkers and k = 3 for locations). The most abundant biomarkers found at most locations were behenic acid, dibutyl phthalate, eicosane, lactic acid, 1-dodecanol, and methyl dehydroabietate.

A conclusion can be drawn from this that most of the samples are of plant origin, and due to the presence of dibutyl phthalate (which is also questionable because of other possible sources of this compound), we are probably dealing with organic residues that have undergone thermal treatment. While fatty acid ratio analysis suggests that meat could be present in the majority of samples, the biomarkers are mostly compounds occurring in plant-based products. In addition, the presence of compounds originating from waxes and resin can indicate that the vessels were sealed or resin was added to enhance the taste. Lactic acid can be used as a biomarker for dairy products. Its presence confirmed the conclusions obtained using the proposed C15:0/C17:0 ratio, suggesting that samples 8 and 9 could contain dairy products. Cholesterol and squalene, which can be considered meat or fish biomarkers, were found in only a few samples. Some compounds, such as gentisic acid, suggest that these samples could contain alcoholic beverages.

Considering the distribution of biomarkers in Figure 4, we can conclude that there is no clear distinction between various settlements in the context of biomarkers. Biomarkers of plant origin and biomarkers of waxes and resins are, even if they appear only in some samples, more or less evenly distributed among the individual samples. This is consistent with the results of the fatty acid ratio analysis. Therefore, we can assume that there were no significant differences in the Neolithic diet between the considered settlements. However, these results should be treated with caution, firstly, because of the uncertain results of the PERMANOVA, and secondly, because the samples come from a very extended historical period.

Our research revealed a varied diet rich in plant and dairy products. The introduction of animal husbandry and grain cultivation may have significantly impacted the chemical composition of organic remains in clay vessels. There is potential to determine differences between the different Neolithic periods, but at the moment, we do not have enough information. For this purpose, all samples should be classified as a specific culture or a specific year, as was the case with samples 24–31 and 32–39.

In summary, we conducted a comprehensive analysis of organic remains in terms of fatty acid content and archaeological biomarkers for a wide range of samples from the Neolithic period. Considering both our results and the archaeological findings cited in the literature, we can determine with high probability that the diet of people inhabiting Polish lands during the Neolithic period was mixed, with a predominant share of plant foods. Similar to our other studies on the Neolithic period in Poland, we can assume that the vessels were sealed or repaired with waxes and resins. Considering the presence of lactic acid and phytanic acid in dairy products, such as milk and its products, they probably had a significant share.

## 3. Materials and Methods

### 3.1. Reagents and Chemicals

Tetracosane (>99.0%), decanoic acid (>99.5%), tridecanoic acid (>99.0%), tetradecanoic acid (>99.5%) and cis-9-octadecenoic acid (>99.0%) were purchased from Fluka/Sigma-Aldrich (Darmstadt, Germany); hexadecanoic acid (>99.0%), heptadecanoic acid (>98.0%) and octadecanoic acid (>99.5%) were purchased from Dr. Ehrenstorfer GmbH (Augsburg, Germany); and icosanoic acid (>99.0%) was purchased from Sigma-Aldrich (Darmstadt, Germany).

HPLC-grade dichloromethane, HPLC-grade methanol, and HPLC-grade n-hexane were purchased from Sigma-Aldrich (Darmstadt, Germany). *N*,*O*-Bis(trimethylsilyl)trifluoroacetamide (BSTFA) and trimethylchlorosilane (TMCS) were purchased from Supelco (Darmstadt, Germany).

### 3.2. Preparation of Experimental Clay Vessels

Archaeologists created nine test vessels using the coil-building method and fired them in a kiln. The following types of food were cooked three times in each vessel: curdled cow’s milk, boiled cow’s milk, boiled goat’s milk, boiled freshwater fish, cooked poultry, cooked beef, cooked pork, cooked leguminous vegetables, and cooked buckwheat. The selection of products was based on the experience and knowledge of archaeologists about the medieval diet of the Slavs. The vessels were then delivered to the laboratory for GC–MS analysis [89].

### 3.3. Characteristics of Archaeological Samples

**Figure 5:**
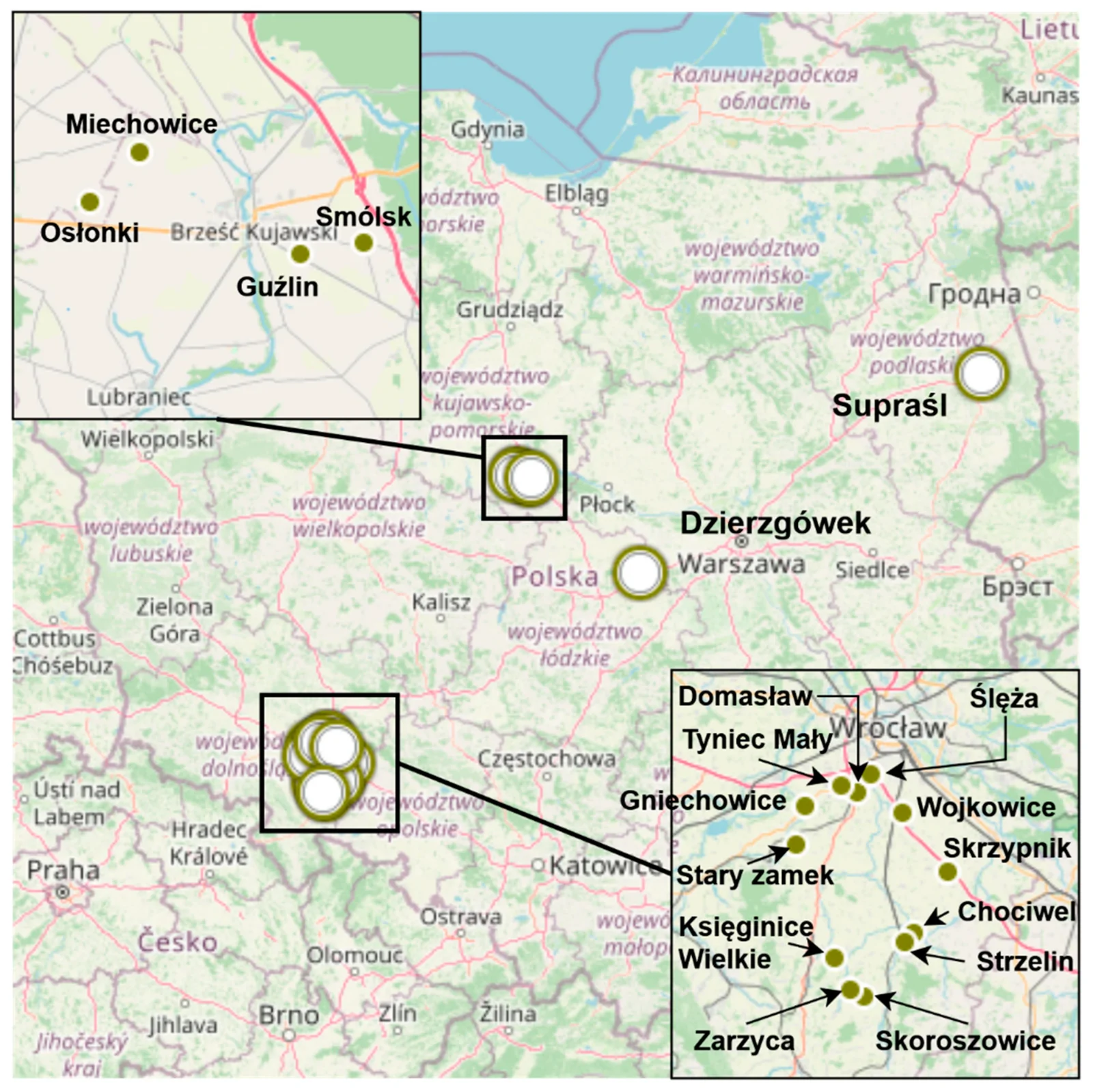
Locations of analyzed samples. Background map ©; OpenStreetMap contributors (ODbL).

The study involved 56 samples of clay vessels collected from various archaeological sites in Poland including Skoroszowice, Strzelin, Chociwel, Ślęża, Wojkowice, Gniechowice, Skrzypnik, Stary Zamek, Zarzyca, Księginice Wielkie, Tyniec Mały and Domasław from the Lower Silesian Province, Guźlin, Osłonki, Smólsk and Miechowice from Kuyavian-Pomeranian Province, Supraśl from Podlaskie Province and Dzierzgówek from Łódź Province (Figure 5). Samples 1–23 and 44–56 originated from the Neolithic period (general), samples 24–31 originated from approximately 5000 BC, samples 32–39 originated from the Lengyel culture (approximately 5000–4000 BC) and samples 40–43 from the period between 2500 and 2000 BC.

### 3.4. Preparation of Archaeological Samples for Analysis

In this study, whole samples of ceramic vessels were collected for analysis. Each sample was cleaned of soil residues and other contaminants using an archaeological brush and deionized water by an archaeologist. Each sample was crushed and ground in a mortar. Five grams of material from each sample was then extracted. Next, they were subjected to Soxhlet extraction (dichloromethane and methanol 2:1 *v*/*v*) for 4 h and then dried in a rotary evaporator. Additionally, 100 μL of tetracosane solution in dichloromethane at 1 mg/mL was added to each sample as an internal standard. Next, each sample was dissolved in 2 mL of hexane, and 0.5 mL of this solution was transferred to a glass vial and evaporated under a stream of nitrogen. The sample was then derivatized by adding 100 µL of BSTFA:TMCS (100:1 *v*/*v*) and heating at 75 °C for 30 min. The vials were filled with 300 µL of hexane and analyzed by gas chromatography coupled with mass spectrometry (GC–MS).

### 3.5. Apparatus Operating Parameters

Chromatographic analysis was performed using a gas chromatograph (GC Agilent Technologies 6890, Santa Clara, CA, USA) coupled with a mass spectrometer (5973 Network Mass Selective Detector, Santa Clara, CA, USA) and equipped with an HP-5MS column (length 30 m, diameter 0.25 mm, film thickness 0.25 µm) (Agilent Technologies, Santa Clara, CA, USA). The choice of column was based on the ability to operate within the boiling point ranges of individual fatty acids and their trimethylsilyl (TMS) derivatives without the risk of packing degradation. The carrier gas (helium) flow rate was 0.9 mL/min. The initial temperature was 60 °C, the temperature increase was 12 °C/min, and the final temperature was 300 °C. The total analysis time was 20 min. All samples were injected by the autosampler. The injection volume was 1 mL and injection mode was splitless. The mass spectrometer operated in electron ionization mode at a potential of 70 eV and sweep rate of 50–550 *m*/*z* (a. m. u.). The ion source temperature was 230 °C and the detector temperature was 150 °C.

In order to determine the risk of accidental sample contamination during sample preparation, blank tests were performed in accordance with the presented analytical procedure. No fatty acids were detected in the obtained chromatograms or they were below the detection limit. In addition, analytical procedures requiring special care, clean reagents, clean glassware, etc., were used.

### 3.6. Method Validation

To validate the analytical procedure, solutions of various reference standard concentrations (10, 20, 40, 50, 80, and 100 μg/mL) were analyzed for the following acids: decanoic acid (C10:0), tridecanoic acid (C13:0), tetradecanoic acid (C14:0), hexadecanoic acid (C16:0), heptadecanoic acid (C17:0), octadecanoic acid (C18:0), icosanoic acid (C20:0), and cis-9-octadecenoic acid (C18:1). This concentration range was selected to cover the most common concentration levels found in archaeological samples. Working solutions for each of the eight acids were prepared by appropriately diluting the stock solutions to a concentration of 1 mg/mL. Six measurements were performed for each tested acid concentration level. Next, the calibration curve was calculated. A correlation coefficient of R^2^ > 0.99 was obtained for each calibration curve.

### 3.7. Data Handling

Chromatographic data were processed using the GC-MSD ChemStation software package, ver. F.01.03.2357 (Agilent Technologies, Santa Clara, CA, USA). The spectra were interpreted using the Enhanced ChemStation Data Analysis software and the NIST08 mass spectrum library (U.S. National Institute of Standards and Technology). Fatty acids were identified when the registered spectra matched the library spectra by at least 80%. The interpretation of the results did not include peaks of compounds originating from the mixture used for derivatization (BSTFA/TMCS) or from column degradation.

### 3.8. Software

Microsoft Excel (Redmond, WA, USA) and Python version 3.13.2 https://www.python.org/ (accessed on 4 February 2025) with the use of libraries Pandas 2.2.3 and NumPy 2.2.3 were used to preprocess the raw data. All calculations and data analysis were performed in R Studio 2025.09.2 https://posit.co/ (accessed on 24 October 2025) using R 4.5.1 https://www.r-project.org/ (accessed on 24 October 2025) with the use of the following packages: ‘*tidyverse*’ for reading data and data manipulation, ‘*stats*’ for ANOVA, ‘*vegan*’ and ‘*pairwiseAdonis*’ for PERMANOVA, ‘*FactoMineR*’ to perform PCA, ‘*factoextra*’ to visualize PCA plots, and ‘*viridis*’ and ‘*pheatmap*’ packages to visualize the heatmap graph.

## 4. Conclusions

By analyzing fatty acid ratios and archaeological biomarkers, we determined that the organic residues in the pottery samples were of a mixed nature, with predominantly plant-based products. We can also assume that the products present in the studied vessels were likely heat-treated. The presence of lactic acid deserves particular attention, as it may suggest that milk or milk products were staples of the Neolithic diet. The presence of wax and resin biomarkers in the studied samples suggests that such substances were used as vascular sealing agents.

We were able to demonstrate that the Neolithic diet was probably similar in all settlements studied. There were no significant differences in dietary habits across settlements (as well as the specific historical periods from which the samples originated) in terms of fatty acid and biomarker composition, but these results should be treated with caution, as data dispersion varies across sample groups. We can also conclude that the people living at that time most likely consumed only local products, and the consumption of animal products was limited.

In our work, for the first time, we have proposed a new proportion of fatty acids (C15:0/C17:0) that can be used to identify milk. The presence of milk or its processed products is confirmed by the presence of lactic acid.

We introduced several novel compounds that, to the best of our knowledge, have not yet been reported in Polish and English literature in the context of the Neolithic diet, including 11-eicosenoic acid, arachidic acid, behenic acid, gentisic acid, *meso*-erythritol, myristyl alcohol (1-tetradecanol), taraxasterol, thymol, benzophenone, caryophyllene, chrysin, and estragole, which can be considered to have originated from plant-based products, and dehydroabietic acid, pimaric acid, (+)-2-bornanone, and eugenol, which can be considered biomarkers of rosin.

Currently, we do not yet have complete information about the cultures and the exact historical origins of all samples, but this research should be continued to determine the variability of diets over time.

## Figures and Tables

**Table 1:** Calculated proportion values for archaeological samples and their interpretation.

Nr	Location	Eerkens Interpretation for Identifying Various Food Products [26]					Olsson Interpretation to Distinguish Fish from Mammals [27]		Hjulström Interpretation to Distinguish Ruminants from Non-Ruminants [28]		Our Interpretation to Identify Dairy Products	
		(C15:0 + C17:0)/C18:0	C16:1/C18:1	C16:0/C18:0	C12:0/C14:0	PSR	C18:0/C16:0	PSR	C17:0/C18:0	PSR	C15:0/C17:0	PSR
1	Skoroszowice	0.36	-	1.20	-	Seeds and nuts	0.84	Mammalian	0.17	Ruminant or milk	1.10	-
2	Skoroszowice	0.35	0.87	1.16	-	Fish, seeds and nuts	0.86	Mammalian	0.16	Ruminant or milk	1.13	-
3	Skoroszowice	0.32	0.25	1.62	-	Terrestrial mammal, seeds and nuts	0.62	Mammalian	0.14	Ruminant or milk	1.37	-
4	Skoroszowice	0.34	0.90	1.06	-	Fish, seeds and nuts	0.95	Mammalian	0.13	Ruminant or milk	1.61	-
5	Skoroszowice	0.21	1.40	1.42	-	Fish	0.70	Mammalian	-	-	-	-
6	Strzelin	0.25	0.59	1.22	0.10	Terrestrial mammal, seeds and nuts	0.82	Mammalian	0.09	Ruminant or milk	1.88	-
7	Chociwel	0.60	0.70	1.48	-	Terrestrial mammal, seeds and nuts	0.67	Mammalian	0.33	Ruminant or milk	0.84	Dairy products
8	Chociwel	0.39	0.96	1.59	0.10	Fish, seeds and nuts	0.63	Mammalian	0.13	Ruminant or milk	2.00	-
9	Ślęza	0.30	0.27	1.54	-	Terrestrial mammal, seeds and nuts	0.65	Mammalian	0.16	Ruminant or milk	0.86	Dairy products
10	Wojkowice	0.25	0.55	1.56	-	Terrestrial mammal, seeds and nuts	0.64	Mammalian	-	-	-	-
11	Wojkowice	0.26	0.57	1.70	-	Terrestrial mammal, seeds and nuts	0.59	Mammalian	-	-	-	-
12	Gniechowice	0.45	1.58	1.72	0.09	Fish	0.58	Mammalian	0.16	Ruminant or milk	1.77	-
13	Gniechowice	0.28	1.40	1.88	0.23	Roots and tubers, seeds and nuts	0.53	Mammalian	-	-	-	-
14	Gniechowice	0.39	1.26	1.63	0.08	Fish	0.61	Mammalian	0.15	Ruminant or milk	1.57	-
15	Skrzypnik	0.25	0.55	1.10	-	Terrestrial mammal, seeds and nuts	0.91	Mammalian	0.11	Ruminant or milk	1.28	-
16	Skrzypnik	0.47	1.16	1.52	-	Fish, seeds and nuts	0.66	Mammalian	0.17	Ruminant or milk	1.72	-
17	Stary Zamek	1.46	0.99	3.20	0.10	-	0.31	Fish	0.41	Ruminant or milk	2.59	-
18	Stary Zamek	0.20	0.26	0.86	0.07	Terrestrial mammal, seeds and nuts	1.17	Mammalian	0.13	Ruminant or milk	0.54	-
19	Zarzyca	0.26	1.14	1.01	0.11	Fish, seeds and nuts	0.99	Mammalian	0.16	Ruminant or milk	0.60	-
20	Zarzyca	0.25	0.56	0.50	0.06	Terrestrial mammal, seeds and nuts	2.00	Mammalian	0.17	Ruminant or milk	0.52	-
21	Zarzyca	0.33	0.21	0.81	0.09	Terrestrial mammal, seeds and nuts	1.23	Mammalian	0.21	Ruminant or milk	0.52	-
22	Zarzyca	0.42	-	2.18	-	Seeds and nuts	0.46	Fish	-	-	-	-
23	Zarzyca	0.32	0.26	0.93	0.06	Terrestrial mammal, seeds and nuts	1.08	Mammalian	0.22	Ruminant or milk	0.44	-
24	Ślęza	0.07	-	0.70	-	Terrestrial mammal	1.42	Mammalian	0.04	Ruminant or milk	0.78	-
25	Ślęza	0.03	-	0.88	-	Terrestrial mammal	1.14	Mammalian	-	-	-	-
26	Ślęza	-	-	-	-	-	-	-	-	-	-	-
27	Guźlin	0.09	-	0.49	-	Terrestrial mammal	2.04	Mammalian	0.08	Ruminant or milk	0.17	-
28	Guźlin	0.08	-	2.44	-	Terrestrial mammal	0.41	Fish	0.08	Ruminant or milk	-	-
29	Guźlin	0.09	-	0.83	-	Terrestrial mammal, seeds and nuts	1.21	Mammalian	0.06	Ruminant or milk	0.48	-
30	Smólsk	-	-	1.73	-	Terrestrial mammal	0.58	Mammalian	-	-	-	-
31	Miechowice	-	-	10.40	1.00	Seeds and nuts, berries	0.10	Fish	-	-	-	-
32	Osłonki	0.57	0.70	3.23	-	Terrestrial mammal, seeds and nuts	0.31	Fish	0.09	Ruminant or milk	5.68	-
33	Osłonki	1.68	1.31	0.96	-	-	1.04	Mammalian	-	-	-	-
34	Osłonki	0.13	-	0.78	-	-	1.28	Mammalian	0.13	Ruminant or milk	-	-
35	Smólsk	-	2.60	0.79	-	Terrestrial mammal	1.27	Mammalian	-	-	-	-
36	Smólsk	1.57	-	2.79	-	-	0.36	Fish	1.16	Ruminant or milk	0.35	-
37	Guźlin	-	-	-	-	-	-	-	-	-	-	-
38	Guźlin	1.69	1.53	0.90	-	-	1.12	Mammalian	0.86	Ruminant or milk	0.96	Dairy products
39	Miechowice	0.42	0.60	0.24	-	Terrestrial mammal, seeds and nuts	4.13	Mammalian	0.42	Ruminant or milk	-	-
40	Supraśl	0.31	0.50	1.84	0.53	Roots and tubers, seeds and nuts	0.54	Mammalian	-	-	-	-
41	Supraśl	0.18	0.33	0.88	0.19	Terrestrial mammal, seeds and nuts	1.14	Mammalian	0.12	Ruminant or milk	0.55	-
42	Supraśl	0.68	0.27	1.49	0.21	Roots and tubers, seeds and nuts	0.67	Mammalian	0.43	Ruminant or milk	0.59	-
43	Supraśl	0.58	0.99	2.38	0.30	Roots and tubers, seeds and nuts	0.42	Fish	0.20	Ruminant or milk	1.84	-
44	Księginice Wielkie	0.33	0.37	1.92	1.38	Roots and tubers, seeds and nuts	0.52	Mammalian	-	-	-	-
45	Księginice Wielkie	1.27	0.44	1.83	0.76	Roots and tubers, seeds and nuts	0.55	Mammalian	0.99	Ruminant or milk	0.28	-
46	Księginice Wielkie	0.19	0.16	0.79	0.35	Terrestrial mammal, seeds and nuts, berries	1.26	Mammalian	0.12	Ruminant or milk	0.63	-
47	Tyniec Mały	0.09	0.31	1.00	0.31	Terrestrial mammal, seeds and nuts, berries	1.00	Mammalian	-	-	-	-
48	Tyniec Mały	0.47	0.63	2.34	0.29	Roots and tubers, seeds and nuts	0.43	Fish	-	-	-	-
49	Domasław	0.94	0.97	3.38	0.25	Roots and tubers, seeds and nuts	0.30	Fish	0.28	Ruminant or milk	2.34	-
50	Domasław	0.57	1.19	3.84	0.22	Roots and tubers, seeds and nuts	0.26	Fish	-	-	-	-
51	Domasław	0.51	1.13	4.27	0.35	Roots and tubers, seeds and nuts	0.23	Fish	-	-	-	-
52	Dzierzgówek	0.43	0.89	3.18	0.34	Roots and tubers, seeds and nuts	0.31	Fish	-	-	-	-
53	Dzierzgówek	0.35	-	2.41	0.32	Seeds and nuts	0.42	Fish	-	-	-	-
54	Dzierzgówek	-	-	1.70	-	-	0.59	Mammalian	-	-	-	-
55	Dzierzgówek	-	-	1.98	-	-	0.51	Mammalian	-	-	-	-
56	Dzierzgówek	-	-	1.95	1.18	Seeds and nuts	0.51	Mammalian	-	-	-	-

C12:0—dodecanoic acid (lauric); C14:0—tetradecanoic acid (myristic); C15:0—pentadecanoic acid (pentadecylic); C16:0—hexadecanoic acid (palmitic); C16:1—cis-9-hexadecenoic acid (palmitoleic); C17:0—heptadecanoic acid (margaric); C18:0—octadecanoic acid (stearic); C18:1—cis-9-octadecenoic acid (oleic).

**Table 2:** A list of biomarkers detected in the tested samples (compounds detected as * TMS derivative, ** 2TMS derivative, *** 3TMS derivative, **** 4TMS derivative).

Name	Sample Numbers
(+)-2-Bornanone	13–14, 18, 20
α-l-Fucopyranose 1,2:3,4-bis(benzeneboronate)	7
β-Tocopherol	22
γ-Sitostenone	7
γ-Sitosterol	5
1,2-Bis(trimethylsilyl)benzene	4–5, 44, 46
(*E*)-11-Eicosenoic acid, *	15, 19, 45, 47
1-Dodecanol *	1–3, 5–23, 40–56
1-Tetradecanol *	2–4, 9–10, 23, 40–56
2,5-Dihydroxybenzoic acid ***	1–2, 5–7, 10–15, 18–21, 23, 40, 43–44, 46–48
Acetovanillone *	1, 11, 15, 19–20, 40, 43, 48
Anthracene	1–2, 4, 6–9, 11–13, 15, 18–21, 41, 47
Arachidic acid *	10
Azelaic acid **	46
Behenic acid *	1–9, 11–21, 23, 40–51, 56
Benzoic acid esters	17, 20, 42, 45–48
Benzoic acid *	6, 12, 14, 17, 44, 49, 52
Benzophenone	3, 5, 7, 9–12, 15, 19, 21–22, 40
Carvacrol *	12, 43
Caryophyllene	3
Cholest-23-ene, (5β)-	47
Cholestan-3-ol, 2-methylene-, (3β,5α)-	41
Cholesterol *	17
Chrysin **	14
Cinnamaldehyde, α-pentyl-	9
d-(−)-Ribofuranose (isomer 2)	2, 10
d-Arabinose ****	44
Dehydroabietic acid *	10
Dibutyl phthalate	1–2, 4–23, 40, 43–44, 47, 49–54
dl-α-Tocopherol	7
Eicosane	1–23, 40–49, 51–56
Estragole	3, 9
Eugenol *	3–4, 7, 9, 13, 19, 40, 44, 47
Fumaric acid, 4-cyanophenyl dodecyl ester	15
Glycerol monostearate **	1, 5, 46
Glycerol ***	1–3, 5–7, 9–12, 14, 16, 18–21, 23, 43–46, 48, 53–54
Isopimaric acid *	10
Isovanillic acid **	1, 10, 19, 23
Isovanillin *	3–5, 7–8, 13–14, 19, 42, 44–46, 50
l-(+)-Rhamnose, benzyloxime (isomer2) ****	20
Lactic Acid **	1–3, 5–16, 18, 20–23, 40, 43–44, 46–47, 49–50, 53
*meso*-Erythritol ****	19
Methyl dehydroabietate	1–3, 6, 8–9, 11–12, 14–21, 23, 40–43, 49–56
Methyleugenol	3, 19
Oxalic acid, isobutyl pentadecyl ester	42
Phenanthrene	3, 10, 48, 50
Phytanic acid	18
Pimaric acid *	10
Salicylic acid **	1
Squalene	4, 6, 11, 50–51
Stigmasta-3,5-diene	9
Stigmastanol *	44–48
Taraxasterol	8
Thymol *	44, 48
Vanillic Acid **	6–7, 12, 15–16, 20, 40, 44, 46
Vanillin *	1–2, 6, 9–12, 15–18, 20–23, 40, 43, 48–49, 51, 53–56
Vanillylmandelic acid ***	12
Vitamin E	7, 9, 21

## Data Availability

The original contributions presented in this study are included in the article/Supplementary Materials. Further inquiries can be directed to the corresponding authors.

## Supplementary Materials

The following supporting information can be downloaded at https://www.mdpi.com/article/10.3390/molecules31132309/s1: File S1: Figure S1: Chromatogram of a reference sample of a vessel with milk; Figure S2: Chromatogram of a reference sample of a vessel with boiled fish; Figure S3: Chromatogram of a reference sample of a vessel with boiled meet; Figure S4: Chromatogram of a reference sample of a vessel with vegetables; Figure S5: Chromatogram of sample nr 1 (Skoroszowice); Figure S6: Chromatogram of sample nr 6 (Strzelin); Figure S7: Chromatogram of sample nr 7 (Chociwel); Figure S8: Chromatogram of sample nr 9 (Ślęża); Figure S9: Chromatogram of sample nr 10 (Wojkowice); Figure S10: Chromatogram of sample nr 12 (Gniechowice); Figure S11: Chromatogram of sample nr 15 (Skrzypnik); Figure S12: Chromatogram of sample nr 17 (Stary Zamek); Figure S13: Chromatogram of sample nr 19 (Zarzyca); Figure S14: Chromatogram of sample nr 40 (Supraśl); Figure S15: Chromatogram of sample nr 44 (Księginice Wielkie); Figure S16: Chromatogram of sample nr 47 (Tyniec Mały); Figure S17: Chromatogram of sample nr 49 (Domasław); Figure S18: Chromatogram of sample nr 52 (Dzierzgówek) (PDF format). File S2: Table S1: Principal component analysis (PCA) scores for the first three principal components (PDF and CSV format). Table S2: Pairwise PERMANOVA (adonis) results comparing organic residue profiles between settlements (PDF and CSV format). File S3: README.md is the data dictionary describing Supplementary Materials.
