# Supplementary material for: Analysis of Organic Residues on Neolithic Pottery in Different Settlements in Poland
Source: Molecules. 2026 Jul 1;31(13):2309. doi: 10.3390/molecules31132309 (PMC13390663; doi:10.3390/molecules31132309)
Supplement: Supplementary file 1 [file molecules-31-02309-s001.zip › File S1 Example Chromatograms.pdf]

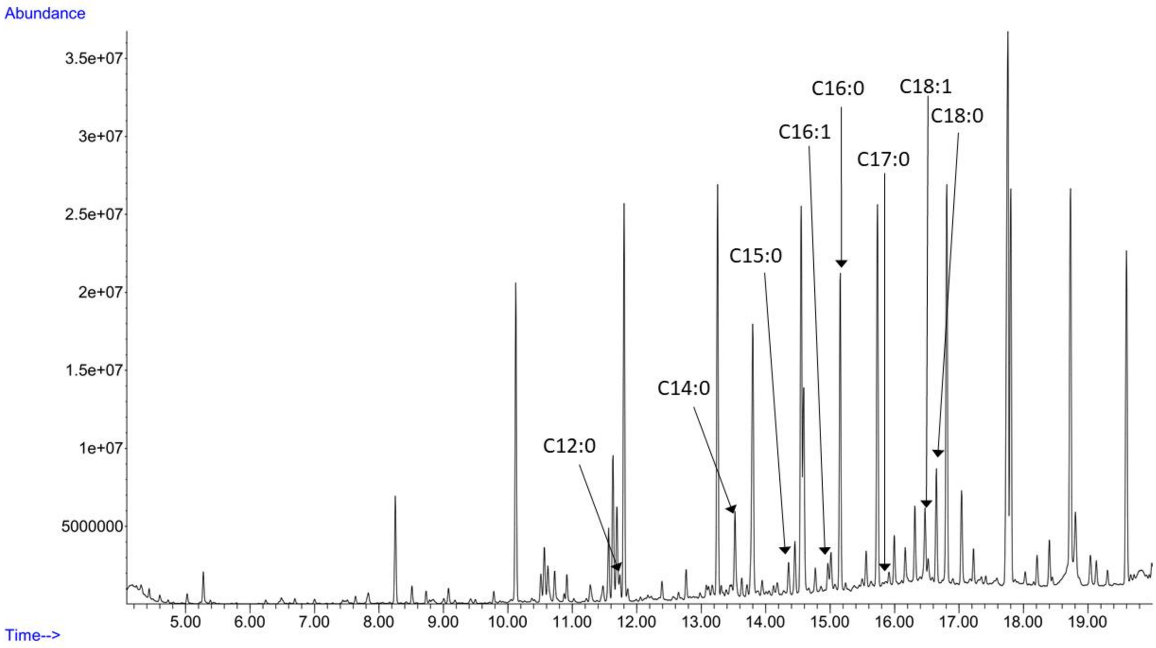

Figure S1. Chromatogram of a reference sample from the vessel with milk.

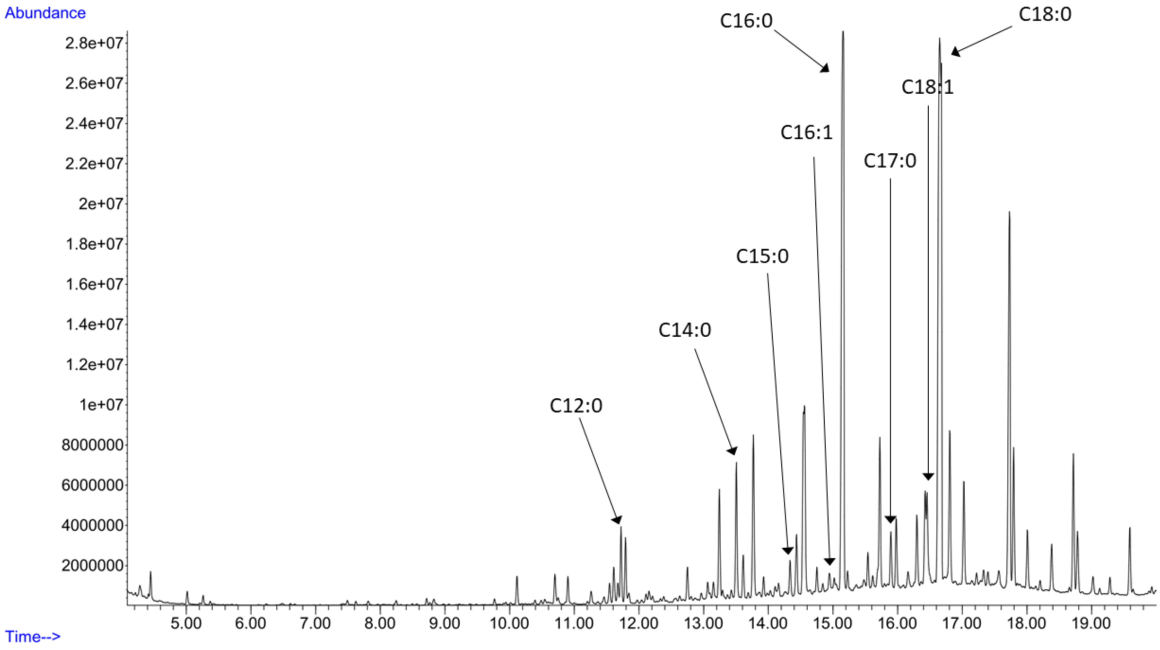

Figure S2. Chromatogram of a reference sample from the vessel with boiled fish.

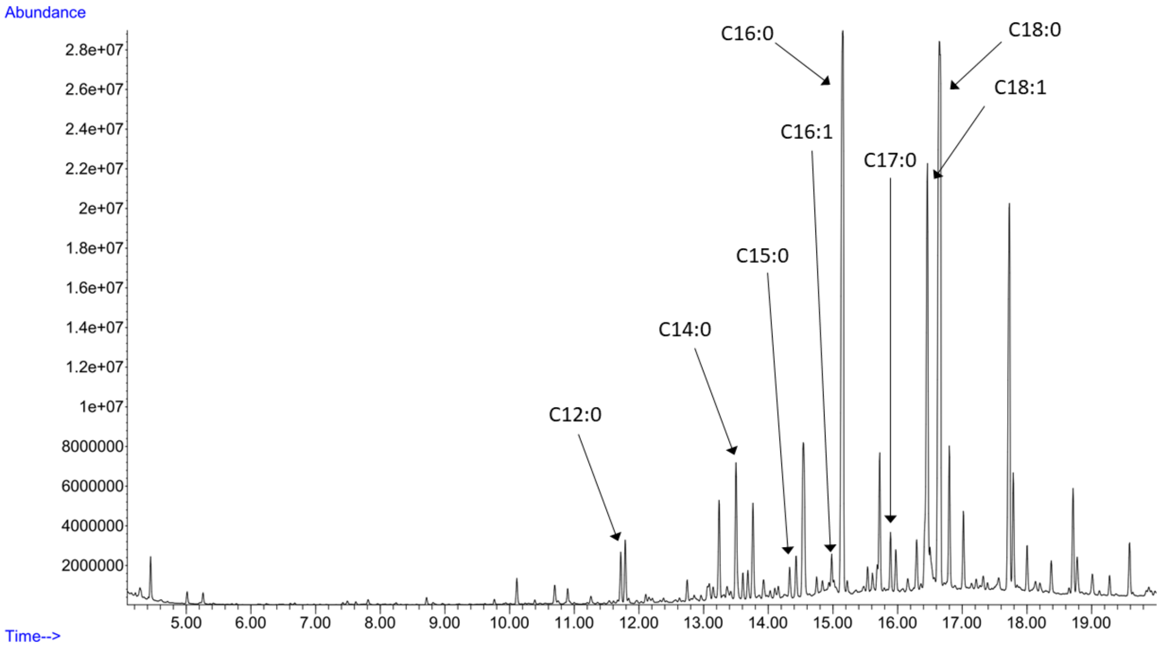

Figure S3. Chromatogram of a reference sample from the vessel with boiled meet.

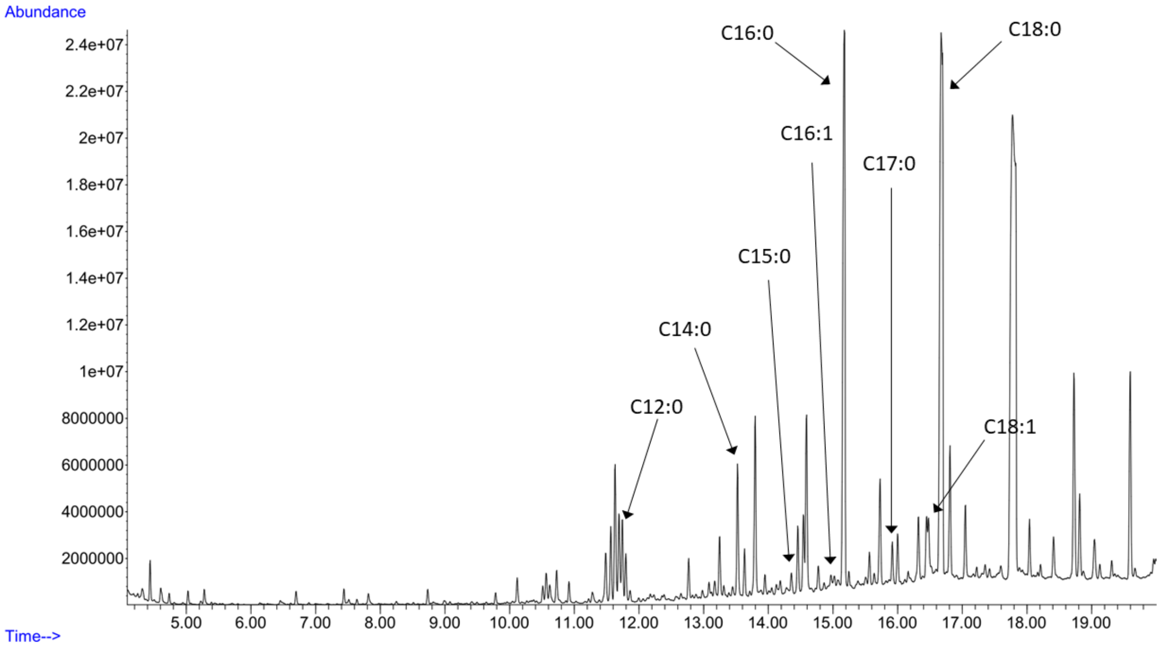

Figure S4. Chromatogram of a reference sample from the vessel with vegetables.

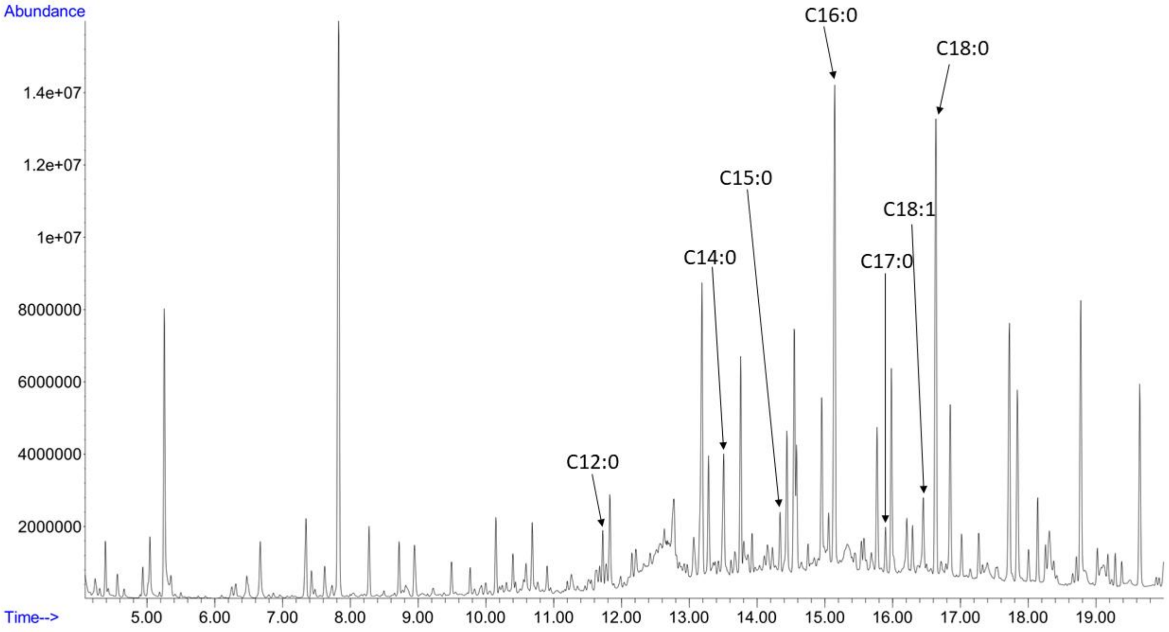

Figure S5. Chromatogram of sample nr 1 (Skoroszowice).

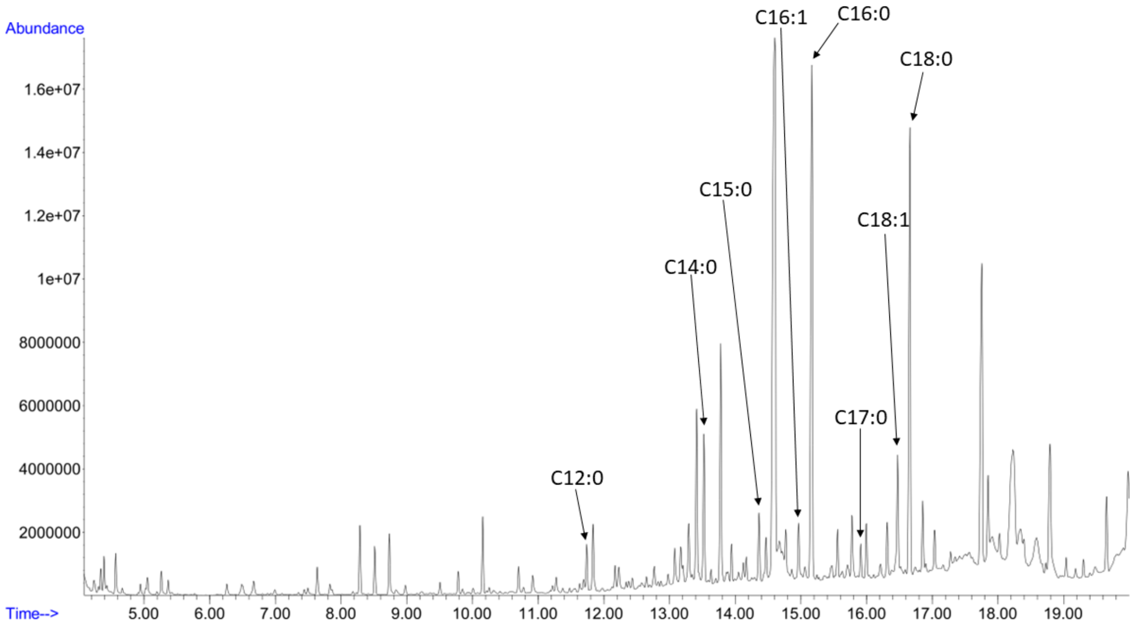

Figure S6. Chromatogram of sample nr 6 (Strzelin).

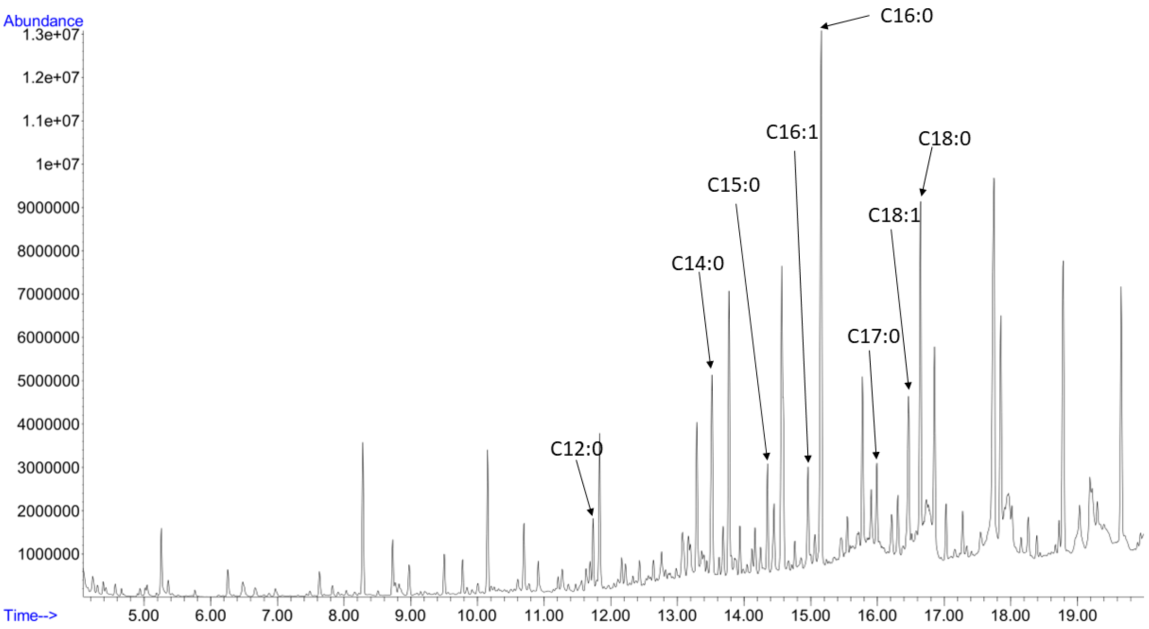

Figure S7. Chromatogram of sample nr 7 (Chociwel).

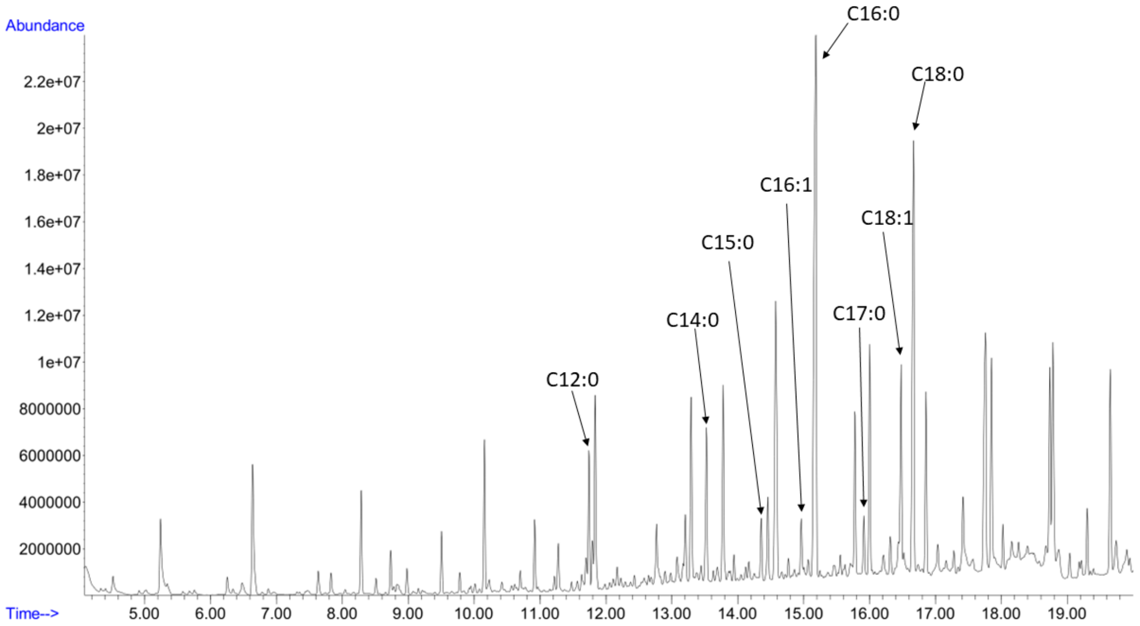

Figure S8. Chromatogram of sample nr 9 (Ślęża).

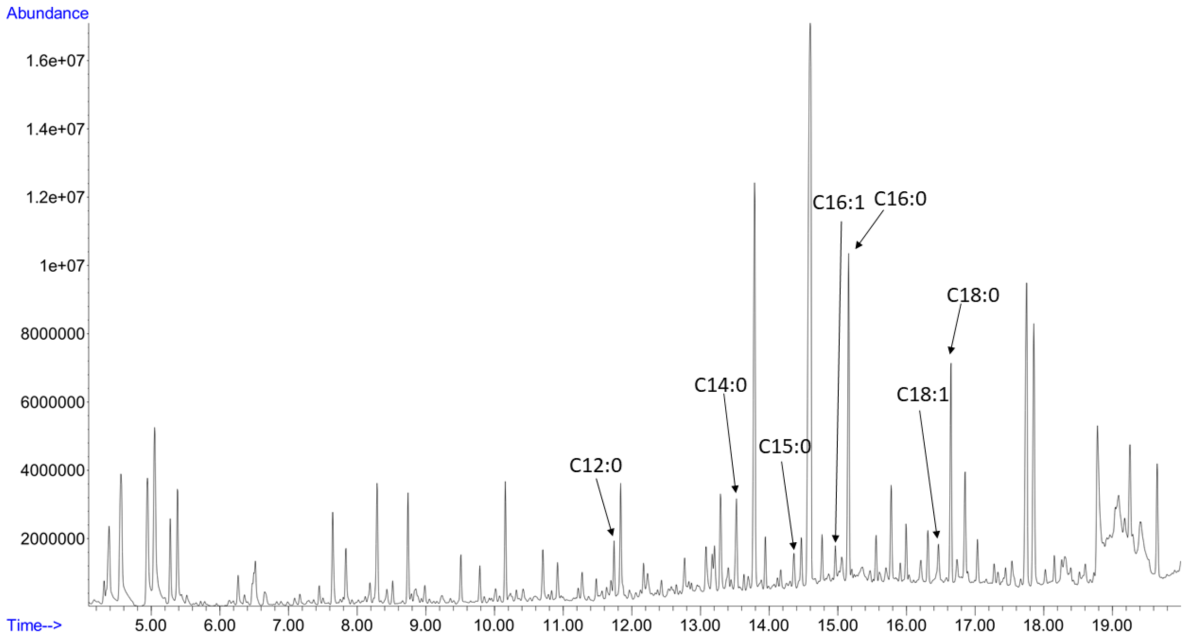

Figure S9. Chromatogram of sample nr 10 (Wojkowice).

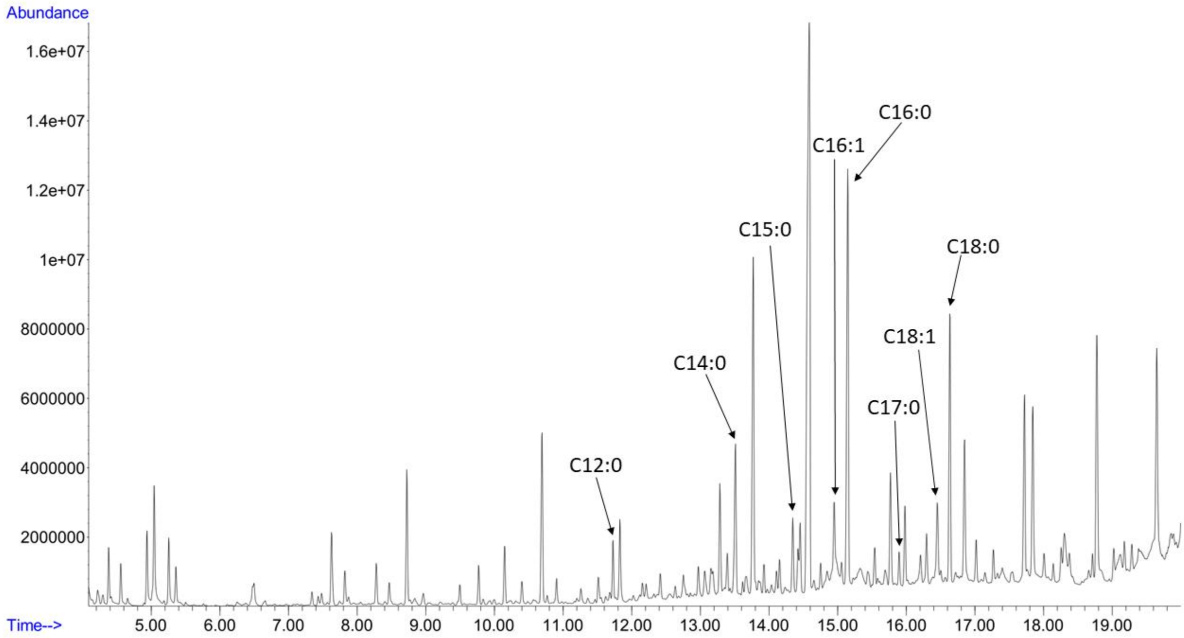

Figure S10. Chromatogram of sample nr 12 (Gniechowice).

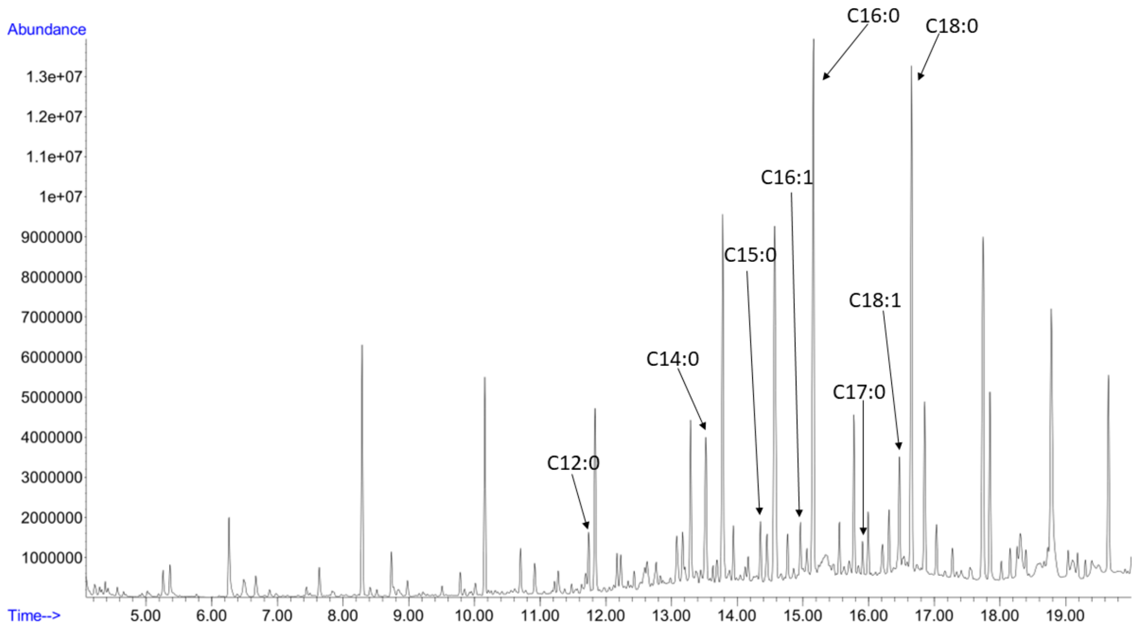

Figure S11. Chromatogram of sample nr 15 (Skrzypnik).

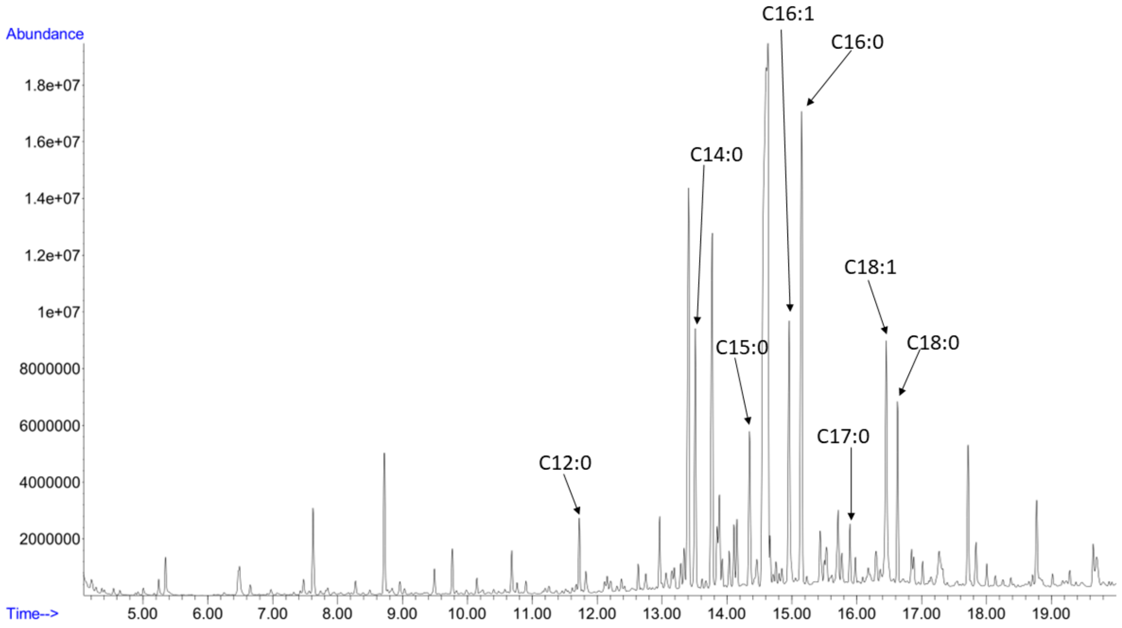

Figure S12. Chromatogram of sample nr 17 (Stary Zamek).

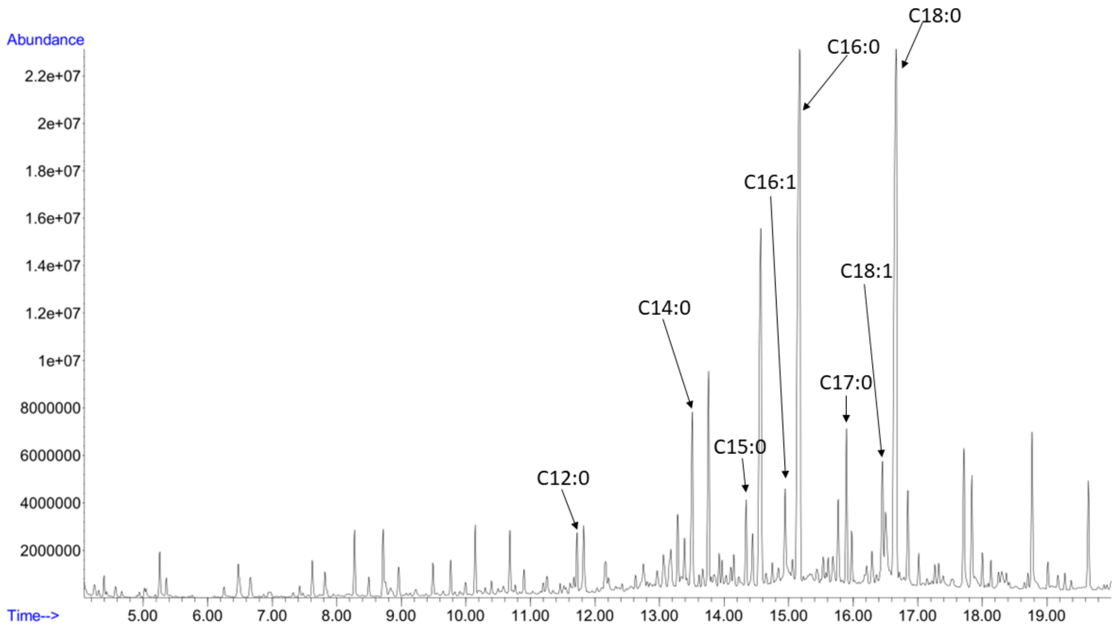

Figure S13. Chromatogram of sample nr 19 (Zarzyca).

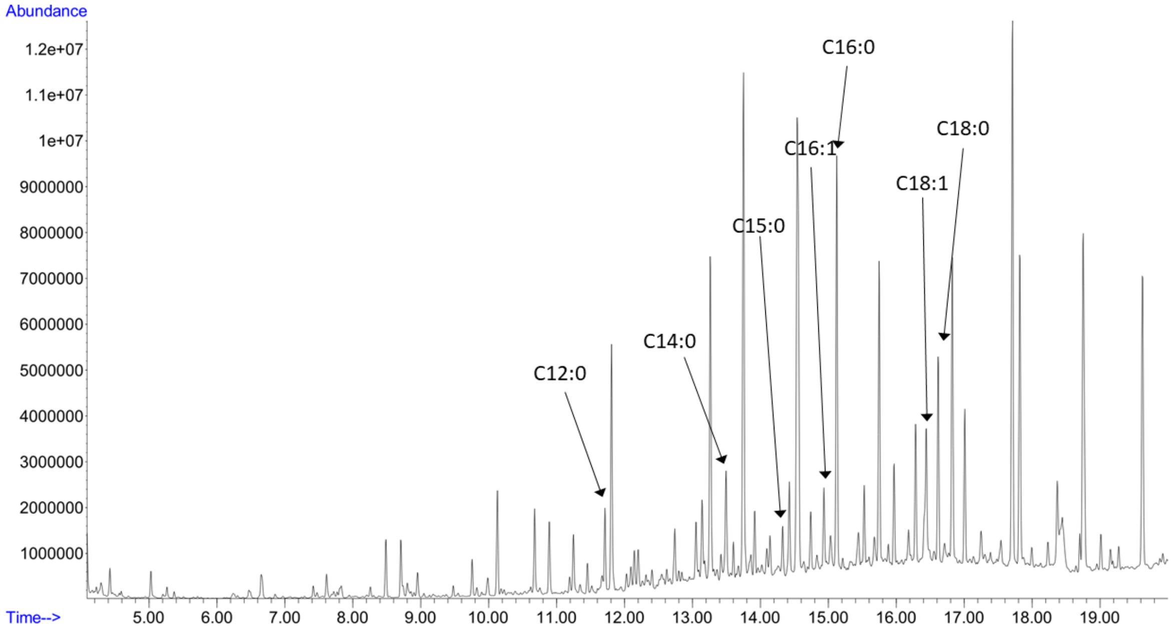

Figure S14. Chromatogram of sample nr 40 (Supraśl).

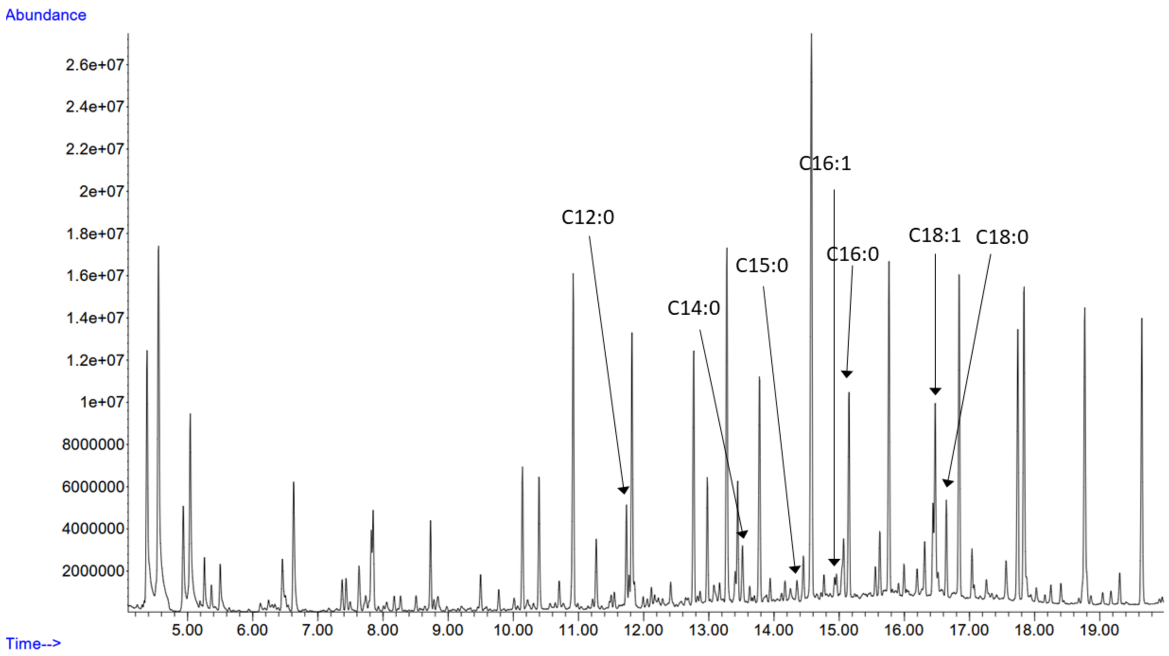

Figure S15. Chromatogram of sample nr 44 (Księginice Wielkie).

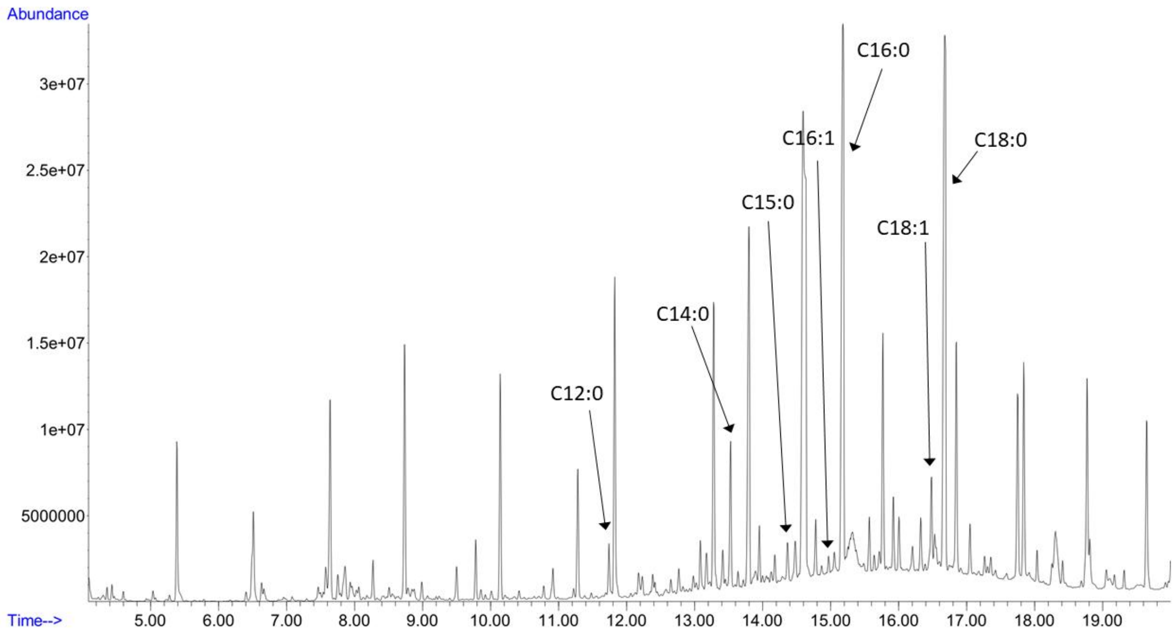

Figure S16. Chromatogram of sample nr 47 (Tyniec Mały).

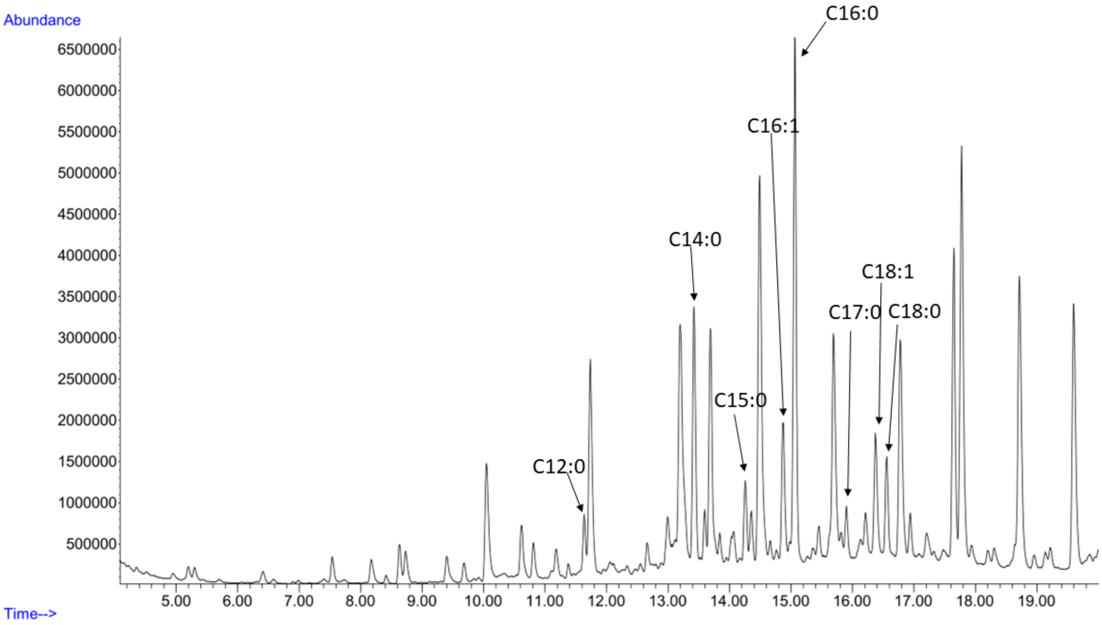

Figure S17. Chromatogram of sample nr 49 (Domasław).

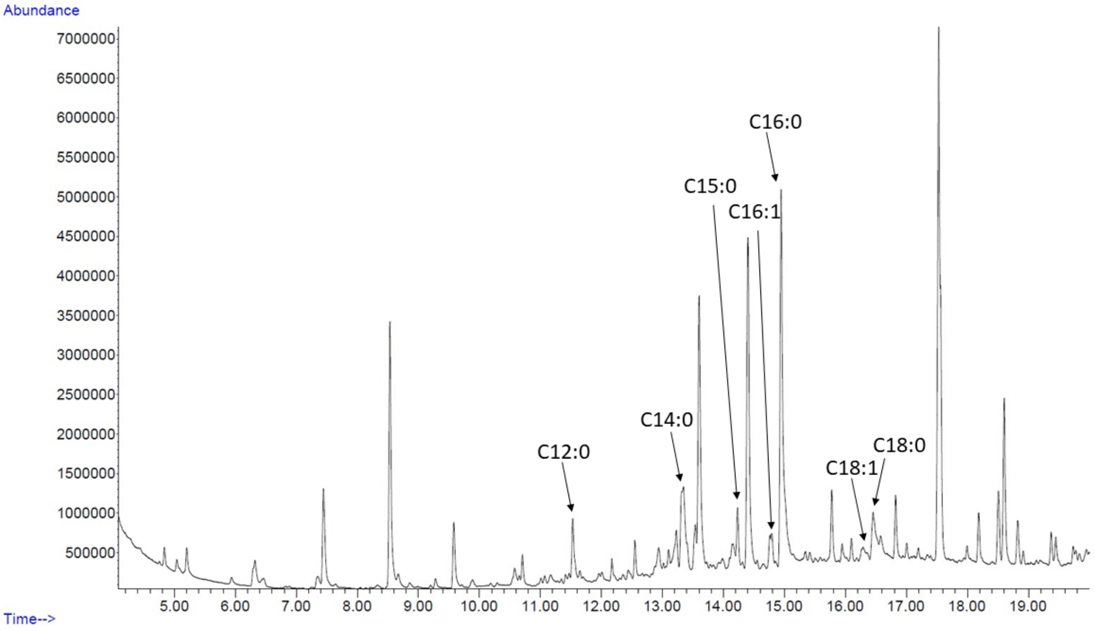

Figure S18. Chromatogram of sample nr 52 (Dzierzgówek).
