# Supplementary material for: Analysis of Organic Residues on Neolithic Pottery in Different Settlements in Poland
Source: Molecules. 2026 Jul 1;31(13):2309. doi: 10.3390/molecules31132309 (PMC13390663; doi:10.3390/molecules31132309)
Supplement: Supplementary file 1 [file molecules-31-02309-s001.zip › File S2.zip!/File S2 Tables.pdf]

Table S1. Principal component analysis (PCA) scores for the first three principal components

PC1 (33.92%)	PC2 (22.88%)	PC3 (16.87%)	Location
-0.602054817646032	-0.406549606077765	-0.128613713895034	Skoroszowice
-0.309300324361893	-0.722256939079345	0.617510870602452	Skoroszowice
0.00454760680570407	-0.0481056860755084	0.399564161306775	Skoroszowice
-0.38797002434676	-0.90028843556575	0.922818564795753	Skoroszowice
-0.42110237448338	0.122675471334116	1.01343644296591	Skoroszowice
-0.286865027232021	-0.34251861637105	0.809673606752477	Strzelin
0.467623102170634	-0.814298685208979	-0.0636057063747031	Chociwel
0.557810586737111	-0.354265857744975	1.11035547648169	Chociwel
-0.202852956549587	-0.0135718157658165	0.122774787071004	Ślęza
-0.58645228495465	0.493253139558485	0.313581996341346	Wojkowice
-0.416445201699897	0.590579260532954	0.350418593194954	Wojkowice
1.00024498226979	-0.542042098582623	1.4535980996254	Gniechowice
0.242540585438681	0.796170492936126	0.686935524692083	Gniechowice
0.630770988979626	-0.375150241211977	1.12933106399329	Gniechowice
-0.684340095807522	-0.514691778230043	0.591499107938832	Skrzypnik
0.597736377136935	-0.730702939960059	1.14290208355655	Skrzypnik
3.67905430527043	-1.05038716334867	0.437687679145341	Stary Zamek
-1.45630109686607	-0.491654747714955	-0.226363689286687	Stary Zamek
-0.644090291414498	-0.604641473104801	0.424774760533662	Zarzyca
-2.46981831976622	-1.71908039377629	-0.302160281980448	Zarzyca
-1.32344852895246	-0.808529324703846	-0.585170765552384	Zarzyca
-0.0196226517919514	0.97257043535124	-0.134674028819611	Zarzyca
-1.0728568802764	-0.654772763252974	-0.504980873830135	Zarzyca
-2.20846495876334	-0.606937737821078	-0.0309478868861036	Ślęza
-2.07548609091802	0.082066139670034	-0.205579052738569	Ślęza
-3.19345622863665	-1.24367962403803	-0.560054675984959	Guźlin
-0.106498739360375	1.30968870816443	-0.0326931892241706	Guźlin
-1.91322374319911	-0.31341559570436	-0.161198465772201	Guźlin
-0.918157335979101	1.04951564314507	0.0413804820851008	Smólsk
2.92011017181271	-0.402515002385752	3.05416078216306	Osłonki
0.546071056983843	-1.75684968115927	-0.0935331717134469	Osłonki
-1.99732932046117	-0.468761585571562	-0.589124974741876	Osłonki
-1.23531958906744	-0.928628422336365	1.94002828747089	Smólsk
3.44164523298899	-2.16869457837149	-2.96388371509247	Smólsk
1.94014134213734	-3.92640958723623	-1.24962214381523	Guźlin
0.00833266211481018	1.51881824983677	-0.58647270858857	Supraśl
-1.36496076847023	-0.229950829612602	-0.316284864340633	Supraśl
0.554280794859599	-0.564466517482403	-1.14510736441955	Supraśl
1.65476446139302	0.22563478775488	0.596835788098339	Supraśl
0.472932415827378	2.9575209502974	-2.11108962847497	Księginice Wielkie
2.61297668747566	-1.06758431088885	-3.52909445758683	Księginice Wielkie
-1.51792324795116	-0.130716755191747	-0.73339805015744	Księginice Wielkie

-1.48633291988755	0.609040274578719	-0.462967274461264	Tyniec Mały
0.576743452826522	1.28228145336687	-0.0994408274283676	Tyniec Mały
3.02692610010899	0.100334231449941	0.67029605874454	Domasław
1.97277435914946	1.71601289402642	0.653161645445104	Domasław
2.22843524008167	2.22464668271951	0.483804568434805	Domasław
1.29267703118894	1.79869583052759	0.195595486068035	Dzierzgówek
0.233350085282719	1.69580266936744	-0.584043655220429	Dzierzgówek
-0.945022899914674	1.02860654746535	0.036024561615897	Dzierzgówek
-0.684424738450076	1.2308328225496	0.0883203522186061	Dzierzgówek
-0.132368171832322	3.09737210894222	-1.88636566495583	Dzierzgówek

Table S2. Pairwise PERMANOVA (adonis) results comparing organic residue profiles between settlements

	pairs	Df	SumsOfSqs	F.Model	R2	p.value	p.adjusted	sig
1	Skoroszowice vs Strzelin	1	0.017999338	0.52795510	0.116599014	0.6666667	0.8888889	
2	Skoroszowice vs Chociwel	1	0.016103889	0.51458360	0.093313228	0.7530000	0.8983158	
3	Skoroszowice vs Ślęza	1	0.126011501	2.79587608	0.317862150	0.0620000	0.4387097	
4	Skoroszowice vs Wojkowice	1	0.102665799	3.74570398	0.428290735	0.0420000	0.3995000	
5	Skoroszowice vs Gniechowice	1	0.073551568	2.48396465	0.292783475	0.1410000	0.5478857	
6	Skoroszowice vs Skrzypnik	1	0.001174759	0.03739463	0.007423408	1.0000000	1.0000000	
7	Skoroszowice vs Stary Zamek	1	0.042815476	0.65905867	0.116460830	0.7310000	0.8983158	
8	Skoroszowice vs Zarzyca	1	0.094073602	1.76558041	0.180796259	0.2000000	0.5773061	
9	Skoroszowice vs Guźlin	1	0.154182924	1.80338779	0.204851568	0.1290000	0.5160000	
10	Skoroszowice vs Smólsk	1	0.153716377	1.66243073	0.216958663	0.1510000	0.5704444	
11	Skoroszowice vs Osłonki	1	0.089849769	0.94499909	0.136069001	0.5030000	0.7435652	
12	Skoroszowice vs Supraśl	1	0.074382906	1.58239627	0.184376976	0.1910000	0.5773061	
13	Skoroszowice vs Księginice Wielkie	1	0.174474270	3.20526030	0.348198769	0.0360000	0.3995000	
14	Skoroszowice vs Tyniec Mały	1	0.143303575	3.40519387	0.405129724	0.0470000	0.3995000	
15	Skoroszowice vs Domasław	1	0.388171529	12.49001988	0.675500619	0.0160000	0.3853333	
16	Skoroszowice vs Dzierzgówek	1	0.422352328	10.92638982	0.577309773	0.0030000	0.3853333	
17	Strzelin vs Chociwel	1	0.015873738	0.78955207	0.441200948	0.6666667	0.8888889	
18	Strzelin vs Ślęza	1	0.083317643	1.24305794	0.383298098	0.5000000	0.7435652	
19	Strzelin vs Wojkowice	1	0.075746628	112.29856833	0.991173763	0.3333333	0.6474366	
20	Strzelin vs Gniechowice	1	0.049266367	2.38617900	0.544022257	0.5000000	0.7435652	
21	Strzelin vs Skrzypnik	1	0.004288796	0.20713219	0.171590315	1.0000000	1.0000000	
22	Strzelin vs Stary Zamek	1	0.013039990	0.06919502	0.064716929	1.0000000	1.0000000	
23	Strzelin vs Zarzyca	1	0.065119413	0.89855345	0.183432408	0.5000000	0.7435652	
24	Strzelin vs Guźlin	1	0.100278358	0.65101212	0.178310040	0.8000000	0.9066667	
25	Strzelin vs Smólsk	1	0.120636090	0.57662848	0.223791860	0.7500000	0.8983158	
26	Strzelin vs Osłonki	1	0.058011066	0.26726741	0.117880849	1.0000000	1.0000000	

27	Strzelin vs Supraśl	1	0.039377463	0.61311653	0.169691879	0.8000000	0.9066667
28	Strzelin vs Księginice Wielkie	1	0.094534380	0.99388435	0.331971523	0.5000000	0.7435652
29	Strzelin vs Tyniec Mały	1	0.089700969	1.21137485	0.547792632	0.3333333	0.6474366
30	Strzelin vs Domasław	1	0.179131519	7.15081684	0.781440276	0.2500000	0.6474366
31	Strzelin vs Dzierzgówek	1	0.204782244	4.73856215	0.542258791	0.1666667	0.5773061
32	Chociwel vs Ślęza	1	0.158142198	3.07754569	0.506379688	0.2000000	0.5773061
33	Chociwel vs Wojkowice	1	0.070779922	6.81255822	0.773051145	0.3333333	0.6474366
34	Chociwel vs Gniechowice	1	0.033727451	1.64797903	0.354558190	0.3000000	0.6474366
35	Chociwel vs Skrzypnik	1	0.009254719	0.45354780	0.184853867	1.0000000	1.0000000
36	Chociwel vs Stary Zamek	1	0.015179717	0.14556868	0.067846198	1.0000000	1.0000000
37	Chociwel vs Zarzyca	1	0.094136098	1.51837178	0.232937278	0.3280000	0.6474366
38	Chociwel vs Guźlin	1	0.157821812	1.30915858	0.246584946	0.4000000	0.6800000
39	Chociwel vs Smólsk	1	0.105761840	0.72353138	0.194313222	0.7000000	0.8897196
40	Chociwel vs Osłonki	1	0.079313115	0.52385341	0.148659250	0.9000000	0.9714286
41	Chociwel vs Supraśl	1	0.034298516	0.64476955	0.138816263	0.7333333	0.8983158
42	Chociwel vs Księginice Wielkie	1	0.103453192	1.47553562	0.329689170	0.2000000	0.5773061
43	Chociwel vs Tyniec Mały	1	0.122238237	2.59656975	0.564892929	0.3333333	0.6474366
44	Chociwel vs Domasław	1	0.170646699	7.29199847	0.708511422	0.1000000	0.4387097
45	Chociwel vs Dzierzgówek	1	0.236908706	6.13851098	0.551106965	0.0440000	0.3995000
46	Ślęza vs Wojkowice	1	0.146955471	3.27228903	0.521705715	0.2000000	0.5773061
47	Ślęza vs Gniechowice	1	0.290251804	6.62124285	0.623396239	0.1000000	0.4387097
48	Ślęza vs Skrzypnik	1	0.121336004	2.35210638	0.439473025	0.3000000	0.6474366
49	Ślęza vs Stary Zamek	1	0.121804918	1.13304989	0.274143773	0.4000000	0.6800000
50	Ślęza vs Zarzyca	1	0.037136998	0.52560010	0.080544332	0.7530000	0.8983158
51	Ślęza vs Guźlin	1	0.019902492	0.16692342	0.032306154	0.9420000	1.0000000
52	Ślęza vs Smólsk	1	0.121415324	0.87907034	0.180171688	0.5000000	0.7435652
53	Ślęza vs Osłonki	1	0.125706658	0.88501234	0.181168906	0.6000000	0.8412371
54	Ślęza vs Supraśl	1	0.166019939	2.54064471	0.336926723	0.1290000	0.5160000
55	Ślęza vs Księginice Wielkie	1	0.180708663	2.22901138	0.357843524	0.2000000	0.5773061

56	Ślęza vs Tyniec Mały	1	0.147591565	2.12768515	0.414940677	0.3000000	0.6474366
57	Ślęza vs Domasław	1	0.585095017	12.70884043	0.760605769	0.1000000	0.4387097
58	Ślęza vs Dzierzgówek	1	0.340186250	6.65038553	0.525706155	0.0120000	0.3853333
59	Wojkowice vs Gniechowice	1	0.088677190	6.33897299	0.678765534	0.2000000	0.5773061
60	Wojkowice vs Skrzypnik	1	0.089443851	8.36701475	0.807080433	0.3333333	0.6474366
61	Wojkowice vs Stary Zamek	1	0.128108461	1.35473306	0.403827379	0.3333333	0.6474366
62	Wojkowice vs Zarzyca	1	0.110328521	1.89854939	0.275209943	0.2880000	0.6474366
63	Wojkowice vs Guźlin	1	0.163679867	1.41475879	0.261278266	0.4000000	0.6800000
64	Wojkowice vs Smólsk	1	0.049219124	0.35232579	0.105098911	1.0000000	1.0000000
65	Wojkowice vs Osłonki	1	0.107934046	0.74475014	0.198878459	0.8000000	0.9066667
66	Wojkowice vs Supraśl	1	0.074322236	1.53757070	0.277661592	0.3333333	0.6474366
67	Wojkowice vs Księginice Wielkie	1	0.094642692	1.48726121	0.331440748	0.3000000	0.6474366
68	Wojkowice vs Tyniec Mały	1	0.029731901	0.79578548	0.284637534	0.6666667	0.8888889
69	Wojkowice vs Domasław	1	0.234644601	13.86364920	0.822102561	0.1000000	0.4387097
70	Wojkowice vs Dzierzgówek	1	0.068275259	1.96714488	0.282345913	0.2080000	0.5773061
71	Gniechowice vs Skrzypnik	1	0.050811652	2.45867979	0.450416564	0.2000000	0.5773061
72	Gniechowice vs Stary Zamek	1	0.070185716	0.91647864	0.234005781	0.6000000	0.8412371
73	Gniechowice vs Zarzyca	1	0.189998361	3.44222127	0.364556302	0.0930000	0.4387097
74	Gniechowice vs Guźlin	1	0.257014097	2.55279852	0.337993727	0.1090000	0.4632500
75	Gniechowice vs Smólsk	1	0.093042651	0.80957361	0.168325444	0.5000000	0.7435652
76	Gniechowice vs Osłonki	1	0.123325745	1.03766280	0.205980996	0.4000000	0.6800000
77	Gniechowice vs Supraśl	1	0.078758039	1.68309164	0.251843268	0.2050000	0.5773061
78	Gniechowice vs Księginice Wielkie	1	0.149823772	2.58846530	0.392878338	0.1000000	0.4387097
79	Gniechowice vs Tyniec Mały	1	0.138925708	3.61340305	0.546375750	0.2000000	0.5773061
80	Gniechowice vs Domasław	1	0.152641612	6.68058952	0.625488837	0.1000000	0.4387097
81	Gniechowice vs Dzierzgówek	1	0.241657914	6.77047070	0.530166104	0.0130000	0.3853333
82	Skrzypnik vs Stary Zamek	1	0.023787036	0.22745484	0.102114230	1.0000000	1.0000000
83	Skrzypnik vs Zarzyca	1	0.086740536	1.39637815	0.218307630	0.2830000	0.6474366
84	Skrzypnik vs Guźlin	1	0.135283912	1.12080630	0.218873012	0.4000000	0.6800000

85	Skrzypnik vs Smólsk	1	0.132481704	0.90508553	0.231770987	0.6000000	0.8412371
86	Skrzypnik vs Osłonki	1	0.065248777	0.43039082	0.125464079	0.8000000	0.9066667
87	Skrzypnik vs Supraśl	1	0.056738048	1.06360145	0.210048413	0.4666667	0.7435652
88	Skrzypnik vs Księginice Wielkie	1	0.136968800	1.94799837	0.393694223	0.3000000	0.6474366
89	Skrzypnik vs Tyniec Mały	1	0.131460346	2.77475694	0.581130511	0.3333333	0.6474366
90	Skrzypnik vs Domasław	1	0.234941826	9.95423496	0.768415502	0.1000000	0.4387097
91	Skrzypnik vs Dzierzgówek	1	0.313115387	8.08790862	0.617967993	0.0430000	0.3995000
92	Stary Zamek vs Zarzyca	1	0.065757467	0.68735319	0.120856428	0.7110000	0.8953333
93	Stary Zamek vs Guźlin	1	0.076971242	0.47326409	0.105798378	0.8000000	0.9066667
94	Stary Zamek vs Smólsk	1	0.073409701	0.36289248	0.107910819	0.9000000	0.9714286
95	Stary Zamek vs Osłonki	1	0.019546543	0.09419147	0.030441383	0.9000000	0.9714286
96	Stary Zamek vs Supraśl	1	0.039967112	0.41946131	0.094912317	0.7333333	0.8983158
97	Stary Zamek vs Księginice Wielkie	1	0.085288967	0.67567234	0.183822789	0.5000000	0.7435652
98	Stary Zamek vs Tyniec Mały	1	0.110176362	0.83943376	0.295634211	0.6666667	0.8888889
99	Stary Zamek vs Domasław	1	0.157292488	1.97807630	0.397357569	0.1000000	0.4387097
100	Stary Zamek vs Dzierzgówek	1	0.244080980	3.37765529	0.403174298	0.0900000	0.4387097
101	Zarzyca vs Guźlin	1	0.045516037	0.42369261	0.057073027	0.6940000	0.8897196
102	Zarzyca vs Smólsk	1	0.130333492	1.10404658	0.155410943	0.3910000	0.6800000
103	Zarzyca vs Osłonki	1	0.072225985	0.59856566	0.090711481	0.7810000	0.9066667
104	Zarzyca vs Supraśl	1	0.072261989	1.04822838	0.130243369	0.3380000	0.6474366
105	Zarzyca vs Księginice Wielkie	1	0.115857353	1.44786191	0.194399671	0.2890000	0.6474366
106	Zarzyca vs Tyniec Mały	1	0.092469047	1.27040805	0.202603728	0.2890000	0.6474366
107	Zarzyca vs Domasław	1	0.465833656	8.22091953	0.578086355	0.0160000	0.3853333
108	Zarzyca vs Dzierzgówek	1	0.449280963	7.76714798	0.492615912	0.0230000	0.3995000
109	Guźlin vs Smólsk	1	0.047292282	0.26854672	0.050971689	0.8350000	0.9385124
110	Guźlin vs Osłonki	1	0.062846574	0.35062466	0.065529668	0.8420000	0.9386230
111	Guźlin vs Supraśl	1	0.153349621	1.40520367	0.189758950	0.2850000	0.6474366
112	Guźlin vs Księginice Wielkie	1	0.155148397	1.18917586	0.192137998	0.3810000	0.6800000
113	Guźlin vs Tyniec Mały	1	0.134016945	0.99984191	0.199974704	0.4666667	0.7435652

114	Guźlin vs Domasław	1	0.484327333	4.72786940	0.486012837	0.0850000	0.4387097
115	Guźlin vs Dzierzgówek	1	0.376690404	4.15270121	0.372349365	0.0360000	0.3995000
116	Smólsk vs Osłonki	1	0.038868874	0.18237085	0.043604659	1.0000000	1.0000000
117	Smólsk vs Supraśl	1	0.092479997	0.75667567	0.131443164	0.6130000	0.8506939
118	Smólsk vs Księginice Wielkie	1	0.093046183	0.61149128	0.132601634	0.7000000	0.8897196
119	Smólsk vs Tyniec Mały	1	0.065224123	0.39733039	0.116953708	0.9000000	0.9714286
120	Smólsk vs Domasław	1	0.209568818	1.78919937	0.309058172	0.2000000	0.5773061
121	Smólsk vs Dzierzgówek	1	0.107720175	1.09308199	0.154105365	0.3980000	0.6800000
122	Osłonki vs Supraśl	1	0.104356273	0.83247887	0.142731570	0.4470000	0.7435652
123	Osłonki vs Księginice Wielkie	1	0.130975154	0.83913086	0.173405284	0.5000000	0.7435652
124	Osłonki vs Tyniec Mały	1	0.093103466	0.54965705	0.154847931	0.7000000	0.8897196
125	Osłonki vs Domasław	1	0.227684235	1.88088717	0.319830515	0.4000000	0.6800000
